# Transcriptome and chromatin alterations in social fear indicate association of MEG3 with successful extinction of fear

**DOI:** 10.1038/s41380-022-01481-2

**Published:** 2022-03-25

**Authors:** Melanie Royer, Balagopal Pai, Rohit Menon, Anna Bludau, Katharina Gryksa, Rotem Ben-Tov Perry, Igor Ulitsky, Gunter Meister, Inga D. Neumann

**Affiliations:** 1 Department of Behavioural and Molecular Neurobiology, Regensburg Center of Neuroscience, University of Regensburg, Regensburg, 93053 Germany; 2 Regensburg Center for Biochemistry, Laboratory for RNA Biology, University of Regensburg, Regensburg, 93053 Germany; 3 Departments of Biological Regulation and Molecular Neuroscience, Weizmann Institute of Science, Rehovot, 76100 Israel

**Keywords:** Molecular biology, Neuroscience, Biochemistry, Psychiatric disorders

## Abstract

Social anxiety disorder is characterized by a persistent fear and avoidance of social situations, but available treatment options are rather unspecific. Using an established mouse social fear conditioning (SFC) paradigm, we profiled gene expression and chromatin alterations after the acquisition and extinction of social fear within the septum, a brain region important for social fear and social behaviors. Here, we particularly focused on the successful versus unsuccessful outcome of social fear extinction training, which corresponds to treatment responsive versus resistant patients in the clinics. Validation of coding and non-coding RNAs revealed specific isoforms of the long non-coding RNA (lncRNA) Meg3 regulated, depending on the success of social fear extinction. Moreover, PI3K/AKT was differentially activated with extinction success in SFC-mice. In vivo knockdown of specific Meg3 isoforms increased baseline activity of PI3K/AKT signaling, and mildly delayed social fear extinction. Using ATAC-Seq and CUT&RUN, we found alterations in the chromatin structure of specific genes, which might be direct targets of lncRNA Meg3.

## Introduction

With a life time prevalence of 8 to 15 % [1] social anxiety disorder (SAD) is among the most common mental disorders and the second most prevalent anxiety disorder [2] with rising numbers worldwide [3–6]. SAD is characterized by excessive fear and avoidance of all social situations. Treatment options for SAD patients are rather unspecific, as underlying molecular mechanisms are unknown. Currently, a combination of cognitive-behavioral therapies with pharmacotherapy is applied, leading to a partial remission of symptoms [7, 8], but there is still a high rate of about 35–45 % of treatment resistance and relapse [8–10].

With the aim to identify molecular mechanisms of SAD, a mouse model of social fear conditioning (SFC) has recently been established to mimic social avoidance behavior — the core symptom of social fear [11]. In the SFC paradigm, mice receive a mild punishment when approaching and investigating a conspecific during social fear acquisition. Subsequently, during social fear extinction training, mice are consecutively exposed to six conspecifics without punishment, which is comparable to exposure therapy in humans. Interestingly, during extinction training, some mice show successful social fear extinction, whereas social fear remains in others, comparable to the human situation. The SFC paradigm generates robust and specific fear of conspecifics without confounding symptoms of general anxiety- or depressive-like behavior. Acute pre-extinction treatment with diazepam or chronic treatment with paroxetine reversed SFC-induced social fear, thus lending predictive validity to the model [11].

Previous studies have shown the involvement of neuropeptides, such as oxytocin and neuropeptide S, in the regulation of extinction of social fear, specifically within the lateral septum [12–14] — a brain region known to play an important role during social stress [15, 16]. However, detailed molecular mechanisms at the level of the transcriptome or chromatin states are still unknown. Given the emerging interest in RNA therapeutics in humans, it seems essential to fill this gap.

In fact, advances in transcriptome mapping have contributed to the identification of various RNAs, many of which are specifically expressed within the brain [17–20]. Different types of non-coding RNAs, such as long non-coding RNAs (lncRNAs), play an important regulatory role for gene expression and in the etiology of psychopathologies including schizophrenia, autism spectrum disorders, Alzheimer’s disease, or anxiety disorders [21–24]. In the present study, we first employed an unbiased approach to investigate SFC-associated alterations in RNA expression. Total RNA-sequencing (RNA-seq) of septal tissue revealed specific isoforms of the lncRNA Maternally Expressed Gene 3 (Meg3) to be differentially expressed in social fear-conditioned (SFC^+^) mice depending on the success of social fear extinction. So far, Meg3 was shown to function as tumor suppressor, to interact with the chromatin-modifying Polycomb repressive complex 2 that introduces trimethylation of histone 3 at lysine 27 (H3K27me3), and to compete for microRNA binding [25–29]. Also, hippocampal Meg3 may regulate non-social learning processes and neuronal plasticity [30, 31]. Altered Meg3 levels have also been identified in blood of patients with Parkinson’s disease, clinically diagnosed psychosis, and schizophrenia, suggesting the possible use of Meg3 as a novel biomarker in human brain disorders [32–34]. Meg3 also has been shown to be regulated in Alzheimer’s and Huntington’s disease using cell culture and various animal models [30, 35]. The *Meg3* locus contains ten exons that are alternatively spliced resulting in several Meg3 isoforms that, in the brain, are almost exclusively found in neurons [36, 37]. However, most studies are not isoform-specific or have mostly focused on the short isoform of Meg3 (~1.9 kb, Meg3-short). In our study, we identified two isoforms containing the alternative long exon 10 (Meg3-ex10) that are dynamically regulated during social fear acquisition and extinction. Using isoform-specific locked nucleic acid (LNA) antisense oligonucleotides (so-called GapmeRs), we revealed the functional relevance of Meg3-ex10 in the context of social fear.

Finally, we identified potential downstream signaling of Meg3-ex10 as well as target gene candidates. We characterized the SFC-related activity of the PI3K/AKT signaling pathway, which was previously shown to be linked to Meg3 in non-social contexts [30, 31, 38], and to be involved in neuronal plasticity, and in learning and memory processes [39–42]. In addition, we also assessed SFC- and Meg3-ex10-dependent alterations of the chromatin status and identified regions with altered histone H3K27me3 modification.

Overall, our study explores previously unknown changes in transcriptome, chromatin accessibility, and histone level modifications in the septum during social fear extinction. Specifically, we identify a spatiotemporal and isoform-specific role of Meg3 during extinction training.

## Material and methods

A detailed list of antibodies, chemicals, and materials used can be found in Supplementary Table 1. Primers are listed in Supplementary Table 2.

### Animals and husbandry

Male CD1 mice (University Clinics of Regensburg, Regensburg, Germany; 8-11 weeks of age at the start of experiment) were kept group-housed under standard temperature- and humidity-controlled conditions with food and water *ad libitum*. Age- and weight-matched male CD1 mice were used as social stimuli in the SFC paradigm. All experimental procedures were performed between 08:00 and 12:00 in accordance with the Guide for the Care and Use of Laboratory Animals of the Government of Unterfranken and the guidelines of the NIH. In all in vivo experiments, the experimenter was blind to the treatment. Group sizes were estimated based on power analysis and results from previous publications [11, 13, 14]. Animals were randomly assigned to experimental groups.

### Social fear conditioning (SFC) paradigm

**Fig. 1:**
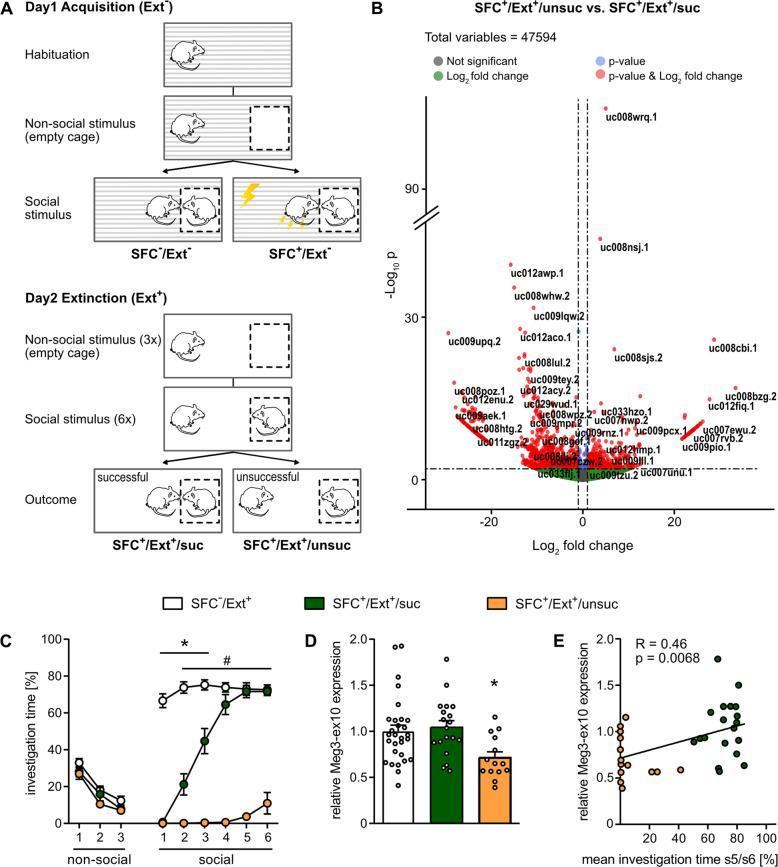
Total RNA-seq revealed dependance of Meg3-ex10 expression on the success of social fear extinction within the mouse septum. **A** Schematic overview of the social fear conditioning (SFC) paradigm. After social fear acquisition (Ext^−^) on day 1 unconditioned (SFC^−^/Ext^−^) and conditioned (SFC^+^/Ext^−^) animals are subjected to the social fear extinction training (Ext^+^) on day 2; SFC^+^ animals are separated according to the success of social fear extinction training resulting in those with successful (SFC^+^/Ext^+^/suc) and those with unsuccessful fear extinction still being fearful at the end of the extinction training (SFC^+^/Ext^+^/unsuc). **B** Volcano plot across data sets for transcript-based differential expression analysis of SFC^+^/Ext^+^/unsuc vs. SFC^+^/Ext^+^/suc mice. The *x*-axis refers to the log_2_ fold change of the RNA transcript in SFC^+^/Ext^+^/unsuc vs. SFC^+^/Ext^+^/suc mice, while the *y*-axis shows the -log_10_ adjusted *p*-value of that comparison. Thresholds are set to *p* < 10^−3^ and |log_2_ fold change | > 1 (doted lines). **C** Investigation time of three non-social and six social stimuli during social fear extinction training of SFC^+^/Ext^+^ mice with successful and unsuccessful fear extinction, and of SFC^−^/Ext^+^ control mice that were used for validating Meg3-ex10 RNA-seq data (*p < 0.05 SFC^−^/Ext^+^ vs. SFC^+^/Ext^+^/suc and SFC^+^/Ext^+^/unsuc; #*p* < 0.05 SFC^+^/Ext^+^/unsuc vs. SFC^−^/Ext^+^ and SFC^+^/Ext^+^/suc, two-way ANOVA, Bonferroni multiple comparison tests). **D** SFC^+^/Ext^+^/unsuc mice expressed lower levels of septal Meg3-ex10 at 90 min after extinction training (*p < 0.05 vs. SFC^−^/Ext^+^ and SFC^+^/Ext^+^/suc, one-way ANOVA, Bonferroni multiple comparison tests). **E** Septal Meg3-ex10 expression positively correlated with the mean investigation time during exposure to social stimulus 5 (s5) and s6 of the social fear extinction training (*p* = 0.0068, Pearson’s *r* = 0.46). Circles: individual data points. Data represent (**C**) mean investigation time ±  SEM, **D** mean fold change + SEM vs. SFC^−^/Ext^+^ and (**E**) relative Meg3-ex10 expression levels plotted against mean investigation time of the fifth (s5) and sixth social stimuli (s6). Group sizes: n(SFC^−^/Ext^+^) = 29, n(SFC^+^/Ext^+^/suc) = 19, n(SFC^+^/Ext^+^/unsuc) = 14.

Mice were single-housed three days before social fear acquisition. The SFC paradigm was performed as previously described [11] (Fig. 1A). Behavioral parameters were manually scored by an observer blind for treatments; social investigation is expressed as percentage of time spent in direct contact with the conspecific during the 3-min exposure. Animals showing less than 30% investigation of the first social stimulus during social fear extinction training were considered as successfully conditioned. For the separate analysis of mice with successful (SFC^+^/Ext^+^/suc) and unsuccessful (SFC^+^/Ext^+^/unsuc) social fear extinction, the mean of the investigation levels for the fifth and sixth social stimuli was calculated with a threshold for SFC^+^/Ext^+^/unsuc set to 45% of investigation. Animals with means > 45% social investigation during the fifth and sixth stimuli were assigned to the successful extinction group (SFC^+^/Ext^+^/suc). Statistical outliers were calculated with the formula “mean ± 2x standard deviation”.

### Microinfusion of antisense LNA GapmeR

In order to down-regulate Meg3-ex10 expression in the lateral septum, GapmeRs were bilaterally microinfused (4 x 70 nl per animal) at two different dorso-ventral positions per hemisphere (from Bregma +0.3 mm anteroposterior, ±0.5 mm mediolateral, –3.4 mm and –3.0 mm dorsoventral) [43] to guarantee their septal distribution. Animals were single-housed and allowed to recover at least for 2 days before behavioral testing. For details see Supplementary Materials.

### RNA and protein extraction, reverse transcription and qRT-PCR

Total RNA and protein were isolated from mouse brain septum using the NucleoSpin miRNA kit (Macherey-Nagel GmbH & Co KG, Düren, Germany) according to the manufacturer’s protocol for animal tissues. 500 ng of total RNA per sample were used for reverse transcription into cDNA using Super Script IV First-strand Synthesis System for qRT-PCR (Invitrogen, Waltham, USA). Relative quantification of RNA levels was performed using PowerUp SYBR Green Master Mix (QuantiFast Qiagen), using Gapdh as housekeeping gene. Primer efficiency for each primer pair was calculated by serial dilution of test cDNA using the Pfaffl method [44].

### Western blot

15 to 30 µg of protein samples were resolved on Criterion™ TGX Stain-Free™ Precast Gels (Bio-Rad, Feldkirchen, Germany) and transferred to a nitrocellulose membrane. Bands were visualized *via* a chemiluminescent reaction with ECL western blot detection reagents (GE Healthcare, United Kingdom; antibodies see Supplementary Table 1). Images were acquired with the ChemiDoc XRS + System (Bio-Rad). All images were analyzed with Image Lab software (Bio-Rad) and abundance of the target protein was normalized to total protein of the lane.

### RNAscope FISH

Brains were fresh-frozen on dry ice and sliced on a cryostat into 10 µm sections, adhered to SuperFrost Plus Slides (VWR). Samples were processed accordingly to the ACD RNAscope Fluorescent Mulitplex V2 Assay manual using a probe for Meg3-ex10 (custom design, #Mm-Meg3-O3, ACDBio, Newark, USA, Supplementary Table 1) and the TSA Plus fluorophore Cy5 (1:5000).

### RNA-seq

Libraries were generated using the Ovation SoLo RNA-Seq System, Mouse (#0501-32, NuGEN Technologies, Leek, Netherlands). Only RNA samples with an RNA integrity number of > 7.4 were used (Agilent 4200 Tape Station System, Agilent High Sensitivity RNA Screen Assay, Agilent Technologies, Santa Clara, California). Paired-end sequencing was performed on HiSeq3000 by the BSF, Vienna, Austria. Raw reads were aligned to the mouse genome (mm10) using HiSAT and StringTie [45] for transcript-based analysis or STAR aligner and HTSeq for count-based analysis [46]. Differential expression analysis was done using DESeq2 for all the analysis.

### ATAC-seq

In order to investigate chromatin accessibility, ATAC-seq was performed as described [47] with minor adjustments for brain tissue. Briefly, frozen brain septum punches were extracted in 500 µl nuclear isolation buffer (see Supplementary Materials). NeuN-positive nuclei were separated by fluorescence-activated cell sorting. Libraries were sequenced with 50 bp paired-end mode on NovaSeq (Illumina, San Diego, USA) and analyzed as previously described [48]. Three replicates were combined for all the analysis in the five groups (SFC^−^/Ext^+^, SFC^+^/Ext^+^/suc, SFC^+^/Ext^+^/unsuc, SFC^+^ control and SFC^+^ Meg3-ex10 knockdown). Reads were aligned to the mouse genome assembly mm10. Each peak was calculated including signals from a surrounding region from ─70 nt to +70 nt.

### uliCUT&RUN

To determine chromatin regions with altered H3K27me3 modifications, the CUT&RUN protocol was performed as described [49, 50] with slight modifications. Frozen brain tissue was resuspended in 1 ml nuclear extraction buffer (Supplementary Materials) for 5 min on ice. The extracted nuclei were pelleted for 3 min at 600 g and prepared for DAPI fluorescence-activated cell sorting to remove debris and collect 50,000 single nuclei for further processing. Libraries were prepared following strictly the protocol from Janssens and Hernikoff, Version 3, 2019 (). Sequencing was performed using 50 bp paired-end mode on NovaSeq (Illumina). Three replicates were combined for all the analysis in the five groups (SFC^−^/Ext^+^, SFC^+^/Ext^+^/suc, SFC^+^/Ext^+^/unsuc, SFC^+^ control and SFC^+^ Meg3 knockdown). Reads were aligned to the mouse genome assembly mm10 using Bowtie2 and peaks were called using MACS2. HOMER was used to quantify and compare the signals to gene bodies and across the ATAC-seq peaks.

### Statistical analysis

For statistical analyses (Prism 8; GraphPad), parametric one-way (factor group) or two-way (factor group x stimulus) analysis of variance (ANOVA), followed by Bonferroni post hoc test, was performed for behavioral and molecular experiments. Non-parametric data was analyzed by Kruskall–Wallis ANOVA on ranks and Dunn’s post hoc test, or two-sided Wilcoxon-rank sum test. Separate parametric *t*-tests between two groups or non-parametric Mann–Whitney U tests were performed. Statistical significance was accepted at *p* < 0.05. Statistical outliers were calculated with the formula “mean ± 2x standard deviation”. Detailed report for all statistical analyses is available in the Supplementary Table 3.

## Results

### Non-coding and coding RNAs are differentially expressed following social fear acquisition and extinction within the septum

We used total RNA-seq to identify regulated RNAs within the septum in response to social fear acquisition or extinction. Brain tissue was sampled from unconditioned (SFC^−^) and conditioned (SFC^+^) male CD1 mice either at 90 min after social fear acquisition, i.e., without extinction (Ext^−^), or after social fear extinction (Ext^+^) (Fig. 1A). As additional controls for social fear acquisition samples, we included animals that were exposed to either the conditioning chamber (*context* group) or to two unpaired shocks (*shock* group*)* only. In this way, changes in transcription initiated by the handling process itself (context) or by physical pain (shock) can be separated from those regulated either by social contact (unconditioned SFC^−^ animals) or by social fear conditioning (conditioned SFC^+^ animals).

We focused mainly on coding and non-coding RNAs that showed differential expression in response to social fear extinction. Initial analysis of our sequencing data indicated dynamic regulation of transcripts between animals exhibiting successful extinction and those that did not. We were interested to carefully examine the process of successful extinction, a factor so far neglected, and the underlying changes in the transcriptome. Subsequently, we increased the number of SFC^+^/Ext^+^ animals to allow the separate expression analyses of animals with successful and unsuccessful extinction (Figure S1A; gene expression data available under GEO: GSE178210). Comparisons were performed on SFC^−^/Ext^+^ vs. SFC^+^/Ext^+^ mice (*n* = 6) (Supplementary Data 1 and 3), as well as on SFC^+^/Ext^+^ mice divided into those with successful fear extinction (SFC^+^/Ext^+^/suc) and those with unsuccessful fear extinction (SFC^+^/Ext^+^/unsuc, *n* = 3 per group) (Supplementary Data 2 and 4, Fig. S1A). As described above, successful and unsuccessful social fear extinction was assessed by the degree of reversal of social fear [11], i.e., with > 45% and < 45% of investigation time of the last two out of six social stimuli, respectively. About 60% of conditioned animals showed successful social fear extinction (SFC^+^/Ext^+^/suc), whereas 40% could not successfully extinguish social fear (SFC^+^/Ext^+^/unsuc). Focusing on the comparison between SFC^+^/Ext^+^/suc and SFC^+^/Ext^+^/unsuc on a transcript-based level, principal component (PC) analysis showed that PC1 explains 23% of the variance, and PC2 15% (Figure S1A). A total of 1638 transcripts (*p* < 0.01, |log_2_ fold change | >1) were regulated; out of these, 652 transcripts were altered with a significantly adjusted *p*-value (<0.01, |log_2_ fold change | >1) (Fig. 1B).

Based on their link to plasticity, learning processes, or neuronal diseases [31, 51–55] and our RNA-seq data analyses, we validated the expression of the non-coding RNAs Nlrp5-ps and Meg3, and of the mRNAs Hcrtr2, Plin4, Sirt1, and Sgk1 (Figure S1) in response to fear extinction in both unconditioned and conditioned animals (for detailed information see GEO: GSE178210, Supplementary Data 1–4). In our gene-based analyses, Hcrtr2 and Sgk1 were significantly regulated after 3 h and 90 min, respectively in unconditioned vs. all conditioned animals (Figure S1C, G, J), whereas the other mRNA candidates and the pseudogene Nlrp5-ps did not show significant changes at 90 min after extinction (Figure S1B, D, E). Interestingly, in transcript-based analyses, specific isoforms of Meg3 containing the alternatively spliced long exon 10 (Meg3-ex10; Figure S2A, B) were found to be regulated depending on the extinction success (Fig. 1C–E). This could be confirmed at 90 min after social fear extinction training, when the relative expression of Meg3-ex10 was significantly lower in SFC^+^/Ext^+^/unsuc mice compared with SFC^+^/Ext^+^/suc and SFC^−^/Ext^+^ mice (Fig. 1D, E). Overall, septal Meg3-ex10 expression levels of all conditioned animals correlated with the extinction success (Fig. 1E) thus confirming the RNA-seq data. Interestingly, the altered expression was isoform-specific for Meg3-ex10 (Figure S2C–F), and specific for the septum, but not found in the dorsal or ventral hippocampus (Figure S2G–J).

Since (i) extinction success shaped the transcription landscape and (ii) the lncRNA Meg3-ex10 was regulated in a social context, which has not been explored before, we further focused on the relevance of Meg3-ex10 in the process of social fear extinction.

### Specific Meg3-ex10 variants are dynamically regulated during social fear and its extinction

**Fig. 2:**
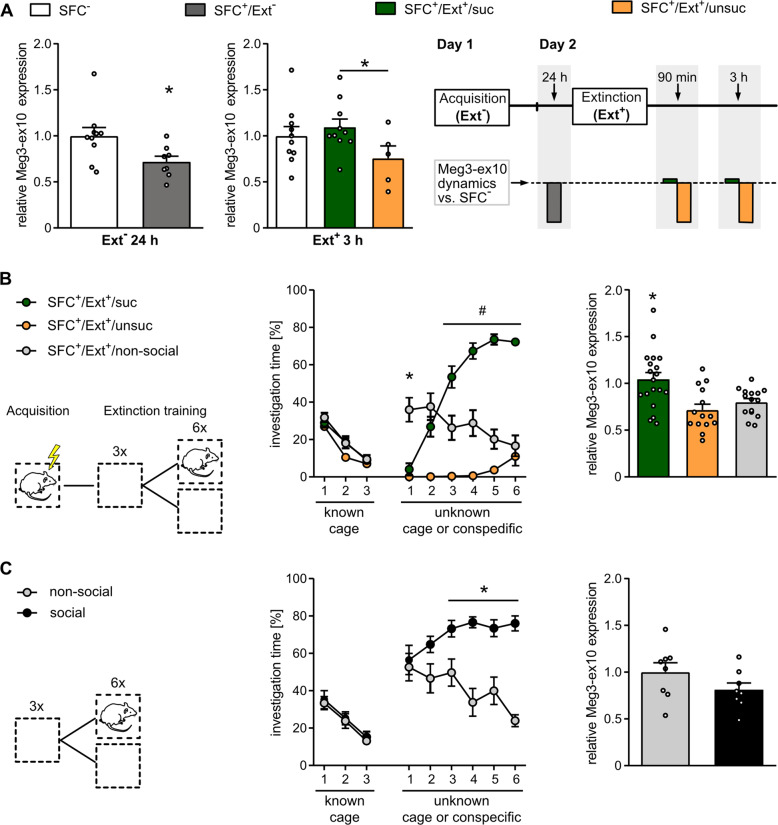
The dynamics of septal Meg3-ex10 expression is linked to the success of the social fear extinction learning processes. **A** Meg3-ex10 levels were downregulated at 24 h after social fear acquisition in SFC^+^/Ext^−^ mice (*p < 0.05 vs. SFC^−^/Ext^−^ mice, unpaired *t*-test, left panel). At 3 h after successful fear extinction, Meg3-ex10 levels returned to higher levels, comparable to SFC^−^/Ext^+^ mice (*p < 0.05, SFC^+^/Ext^+^/suc vs. SFC^+^/Ext^+^/unsuc; separate statistics: unpaired *t*-test, middle panel). Data represent mean ± SEM; Group sizes: Ext^−^ 24 h: n(SFC^−^/Ext^−^) = 10; n(SFC^+^/Ext^−^) = 8; Ext^+^ 3 h: n(SFC^−^/Ext^+^) = 10; n(SFC^+^/Ext^+^/suc) = 10; n(SFC^+^/Ext^+^/unsuc) = 5. Scheme of time points used for investigating Meg3-ex10 levels during the SFC paradigm (right panel, upper part) and schematic overview of expression dynamics in mice with successful (SFC^+^/Ext^+^/suc) and unsuccessful (SFC^+^/Ext^+^/unsuc) fear extinction relative to corresponding SFC^-^ group (right panel, lower part). **B** After social fear acquisition, SFC^+^ mice were either exposed to unknown six objects (empty cages; SFC^+^/Ext^+^/non-social) or to six unknown conspecifics during the extinction training (scheme left panel). The data were combined with data of SFC^+^/Ext^+^/suc and SFC^+^/Ext^+^/unsuc mice shown in Fig. 1E as they were collected during the same experiments. SFC^+^/Ext^+^/non-social mice investigated the first unknown object longer than SFC^+^/Ext^+^/suc and SFC^+^/Ext^+^/unsuc the first unknown social stimulus (*p < 0.05 vs. SFC^+^/Ext^+^/suc and SFC^+^/Ext^+^/unsuc, two-way ANOVA, Bonferroni multiple comparison tests, middle panel) indicating preference for novelty and no fear of SFC^+^ mice towards empty cages. From the third stimulus on, SFC^+^/Ext^+^/suc mice explored presented social stimuli longer than SFC^+^/Ext^+^/unsuc and SFC^+^/Ext^+^/non-social mice their respective social or non-social stimuli (#*p* < 0.01, two-way ANOVA, Bonferroni multiple comparison tests) indicating successful social fear extinction. Group sizes: *n* = 14–19. Septal Meg3-ex10 levels were normalized to SFC^−^/Ext^+^ mice (not shown). Meg3-ex10 levels were similar in SFC^+^/Ext^+^/non-social and SFC^+^/Ext^+^/unsuc mice at 90 min after extinction training and significantly lower than Meg3-ex10 levels of SFC^+^/Ext^+^/suc mice (*p < 0.05 SFC^+^/Ext^+^/suc vs. SFC^+^/Ext^+^/non-social and SFC^+^/Ext^+^/unsuc, two-way ANOVA, Bonferroni multiple comparison tests; right panel). **C** Naïve mice, which were not subjected to social fear acquisition (scheme left panel), investigated unknown social stimuli more than mice subjected to unknown non-social stimuli (empty cages), indicating naturally occurring social preference behavior (*p < 0.05, two-way ANOVA, Bonferroni multiple comparison tests; middle panel). Septal Meg3-ex10 expression did not differ between naïve mice repeatedly exposed to either social stimuli or non-social stimuli (group sizes: *n* = 8; right panel). Data represent mean investigation levels ± SEM for behavioral data (2B, C middle panels) and mean fold change + SEM of Meg3-ex10 levels (2 A left and middle panels, 2B-C right panel). Circles: individual data points.

To generate an overview of the temporal dynamics of septal Meg3-ex10 expression during the SFC paradigm, we additionally quantified Meg3-ex10 levels at 24 h after social fear acquisition and at 3 h after extinction training (Fig. 2A). At 24 h after acquisition, i.e., immediately before extinction training, SFC^+^/Ext^−^ mice revealed significantly downregulated levels of Meg3-ex10 compared with SFC^−^/Ext^−^ (Fig. 2A left panel). At 3 h after extinction training, Meg3-ex10 levels remained low in SFC^+^/Ext^+^/unsuc compared with SFC^+^/Ext^+^/suc mice (Fig. 2A middle panel), thus confirming initial results of Meg3-ex10 levels at 90 min after social fear extinction (Fig. 1D). In summary, the schematic overview presented in Fig. 2A (right panel) visualizes that social fear acquisition results in low septal Meg3-ex10 levels after 24 h, which are restored to a comparable level found in unconditioned mice (SFC^-^/Ext^+^) after successful, but not after unsuccessful fear extinction. These results indicate that (i) Meg3-ex10 expression is negatively linked to social fear, and (ii) social fear acquisition and successful extinction have opposing effects on Meg3-ex10 regulation (Fig. 2A).

To test whether learning processes are necessary for the upregulation of Meg3-ex10 in SFC^+^/Ext^+^/suc mice, we included an additional group of SFC^+^ mice, which was prevented from extinguishing social fear during extinction training, as only objects (empty cages), but not social stimuli, were presented (SFC^+^/Ext^+^/non-social) (Fig. 2B, left and middle panel). The data of SFC^+^/Ext^+^/suc and SFC^+^/Ext^+^/unsuc mice shown in Fig. 1C, D were combined with the SFC^+^/Ext^+^/non-social group, as experiments were performed in parallel. Interestingly, the lack of presentation of social stimuli prevented the restoration of Meg3-ex10 levels in the SFC^+^/Ext^+^/non-social group, which remained at low levels found in SFC^+^/Ext^+^/unsuc mice and significantly differed from the SFC^+^/Ext^+^/suc group 90 min after the last stimulus presentation (Fig. 2B right panel) indicating the importance of extinction learning in regulating septal Meg3-ex10.

As SFC^+^/Ext^+^/suc mice also faced more direct social interaction during the successful social fear extinction training, we separately addressed social interaction as a responsible factor for Meg3-ex10 regulation. Hence, additional groups of mice, deprived of social interaction for three days (identical to all SFC mice prior to acquisition training) and not subjected to social fear conditioning (SFC-naïve) were included. These SFC-naïve mice were exposed to the SFC extinction protocol, but one group was presented six unknown social stimuli (conspecifics) and the other six unknown non-social stimuli (empty cages) (Fig. 2C, left panel). Although naturally occurring social preference behavior, i.e., higher investigation times of the social versus the non-social stimuli [56], was found (Fig. 2C, middle panel), septal Meg3-ex10 levels did not differ between the social and non-social group of naïve mice at 90 min after the last stimulus presentation (Fig. 2C right panel). Thus, repeated social interaction per se does not influence Meg3-ex10 expression.

### Meg3-ex10 knockdown prior to social fear acquisition, but not thereafter, delays social fear extinction

We next asked, whether knockdown of septal Meg3-ex10 levels impacts on behavior during SFC. For these loss-of-function studies, different antisense LNA GapmeRs targeting only Meg3-ex10 were tested with GapmeR5 (0.05 nmol per hemisphere) being the most efficient one (Figure S3). An incubation time of 72 h was chosen to allow mice to recover from anesthesia and surgery.

**Fig. 3:**
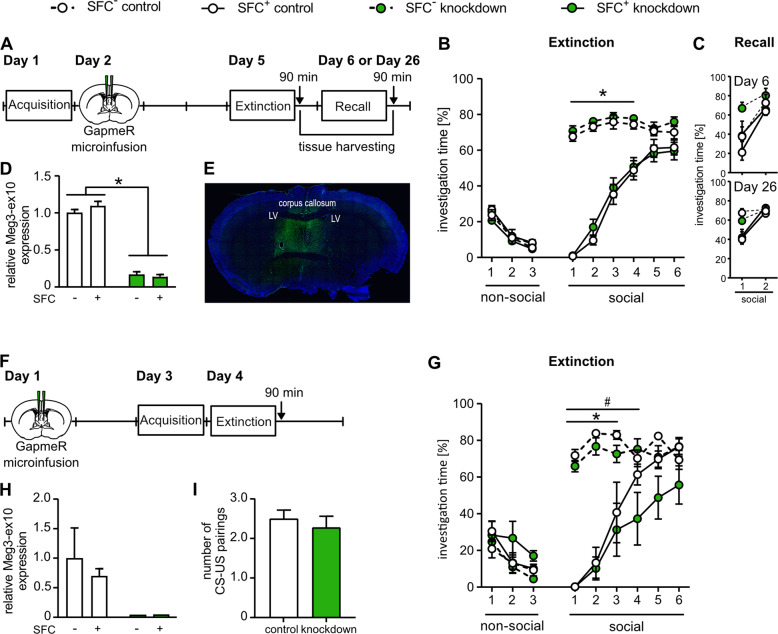
Meg3-ex10 loss-of-function resulted in delayed social fear extinction. **A**, **F** Schematic timeline for behavioral testing and microinfusion of antisense LNA GapmeRs targeting Meg3-ex10. Antisense LNA GapmeRs were bilaterally microinfused into the septum (0.05 nmol); extinction training was performed 72 h thereafter. **B** Meg3-ex10 knockdown induced 24 h after acquisition and 72 h prior to social fear extinction did neither affect social fear expression (*p < 0.005 SFC^+^ control/knockdown vs. SFC^−^ control/ knockdown, respectively) nor the dynamics of social fear extinction (*p* = 0.9998 SFC^+^ control vs. SFC^+^ knockdown). n(SFC^−^ control/SFC^−^ knockdown) = 21/19, n(SFC^+^ control/SFC^+^ knockdown) = 33/34. **C** Subgroups were subjected to either short-term (on day 6) or long-term (on day 26) recall. Day 6: n(SFC^−^ control/SFC^−^ knockdown) = 4/2, n(SFC^+^ control/SFC^+^ knockdown) = 10/7; Day 26: n(SFC^−^ control/SFC^−^ knockdown) = 6/6, n(SFC^+^ control/SFC^+^ knockdown) = 9/14. **D** Mice were sacrificed at 90 min after the last behavioral assessment to investigate septal Meg3-ex10 expression levels, or to visualize the infusion site and GapmeR distribution within the septum (not shown). **E** Distribution of FAM-labeled antisense LNA GapmeRs within the septum 23 days after microinfusion. **G** Social fear memory consolidation was not impaired by Meg3-ex10 knockdown, as similarly low investigation levels of the first social stimulus during social fear extinction training were found in SFC^+^ control and SFC^+^ knockdown mice. However, in SFC^+^ knockdown mice social fear extinction was delayed by Meg-ex10 knockdown, as the significant difference in investigation times compared to SFC^−^ mice remained until exposure to the fourth social stimulus (*p < 0.05 SFC^+^ control vs. SFC^−^ control, # *p* < 0.05 SFC^+^ knockdown vs. SFC^−^ knockdown, two-way ANOVA, Bonferroni multiple comparison tests). *n* = 5–7. **H** Meg3-ex10 knockdown induced before the acquisition training was either verified by controlling the infusion site and the GapmeR distribution (not shown) or by measuring relative Meg3-ex10 levels at 90 min after the social fear extinction training. **I** Meg3-ex10 knockdown 48 h prior to social fear acquisition did not affect social fear acquisition, as SFC^+^ control and knockdown animals required a similar number of pairings between the conditioned stimulus (CS, unknown conspecific) and an unconditioned stimulus (US, 0.7 mA foot shock) to acquire social fear during social fear acquisition. Data represent mean fold change + SEM normalized to corresponding control group or mean investigation time ± SEM during social fear extinction or recall.

In the first Meg3-ex10 loss-of-function experiments, GapmeR microinfusion was performed at 24 h after social fear acquisition and 72 h prior to social fear extinction (Fig. 3A). Both SFC^+^ control and SFC^+^ knockdown mice displayed social fear at the beginning of the extinction training (Fig. 3B), indicated by significantly reduced investigation times of the social stimuli compared to those in the respective SFC^−^ control groups. However, the dynamics of fear extinction did not differ between the two treatment groups (Fig. 3B) indicating that Meg3-ex10 knockdown after acquisition and prior to extinction does not affect social fear extinction. Social motivation was not affected by Meg3-ex10 knockdown, as indicated by an about 12-fold higher investigation of the social vs. non-social stimuli in both SFC^−^ knockdown and SFC^−^ control mice (Fig. 3B).

To investigate potential effects of Meg3-ex10 knockdown on extinction memory formation and consolidation, mice were exposed to either short-term (24 h) or long-term (21 days) social fear recall (Fig. 3C). Again, no differences in social investigation time were detected between control and knockdown groups at either time point. Efficient Meg3-ex10 knockdown at 90 min after the last behavioral testing was verified by qRT-PCR or fluorescence imaging revealing GapmeR distribution within the septal area, even 25 days after microinfusion (Fig. 3D, E).

To test whether Meg3-ex10 knockdown affects acquisition learning, Meg3-ex10 knockdown was induced before social fear acquisition (Fig. 3F). However, the same number of foot shocks had to be applied to SFC^+^ control and knockdown mice to induce social fear, indicating no effect of Meg3-ex10 knockdown on social fear acquisition (Fig. 3I). Moreover, social fear memory consolidation was not affected by Meg3-ex10 knockdown prior to acquisition, as both SFC^+^ treatment groups expressed social fear at the beginning of the extinction training to the same extend. However, the dynamics of social fear extinction was delayed in SFC^+^ knockdown mice, as they still showed reduced investigation times until the fourth stimulus compared to SFC^−^ knockdown, whereas SFC^+^ control mice showed similar investigation levels as the SFC^-^ control group from the fourth stimulus on (Fig. 3G). Meg3-ex10 knockdown was verified by qRT-PCR (Fig. 3H) or distribution of FAM-labeled GapmeRs by fluorescence imaging (not shown).

In summary, our stepwise manipulation of Meg3-ex10 during the SFC paradigm showed that only Meg3-ex10 knockdown prior to social fear acquisition delays social fear extinction without affecting social fear acquisition or extinction memory consolidation.

### The PI3K/AKT signaling pathway is differentially activated depending on successful extinction of social fear and on Meg3-ex10 expression

Due to the neuronal effects of the PI3K/AKT signaling pathway and its link to Meg3 [30, 31, 57, 58], we selectively assessed the activation of this pathway in the septum (i) depending on the success of social fear extinction and (ii) after local Meg3-ex10 knockdown.

**Fig. 4:**
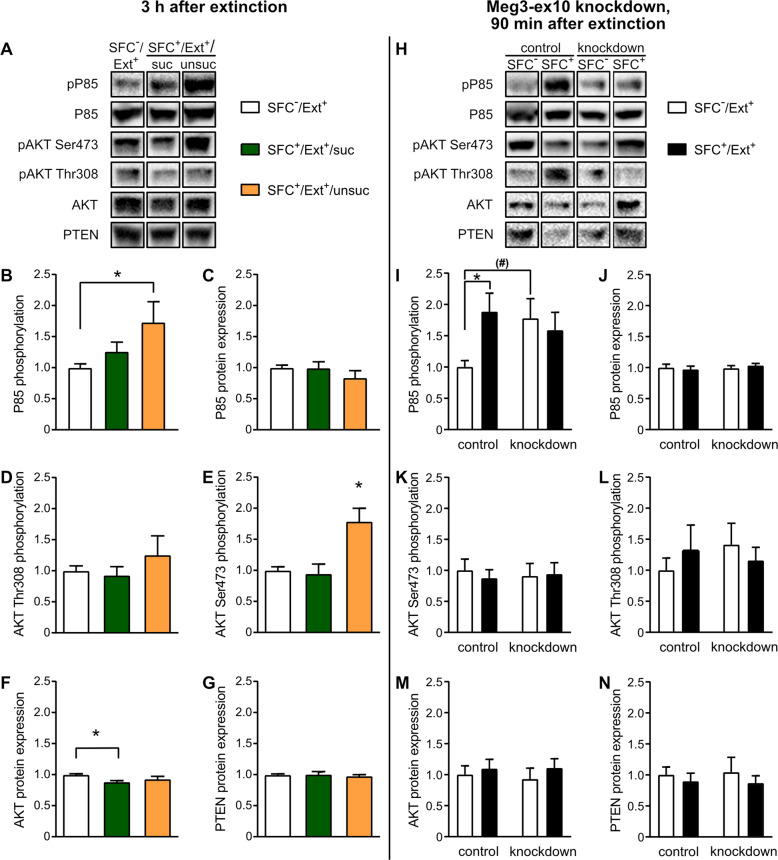
The activity of the PI3K/AKT signaling pathway within the septum differs between mice with successful and unsuccessful extinction, and is altered by Meg3-ex10 knockdown. **A**–**G** Protein and phosphorylation levels were measured at 3 h after social fear extinction training in unconditioned mice (SFC^−^/Ext^+^), and in conditioned mice with successful (SFC^+^/Ext^+^/suc) and unsuccessful (SFC^+^/Ext^+^/unsuc) social fear extinction. **B** SFC^+^/Ext^+^/unsuc mice displayed higher phosphorylation levels of P85 than SFC^−^/Ext^+^ (pP85, *p < 0.05) and (**E**) higher phosphorylation levels of AKT Ser473 than SFC^−^/Ext^+^ and SFC^+^/Ext^+^/suc mice (*p < 0.05, one-way ANOVA, Bonferroni multiple comparison tests). **F** Total AKT protein was significantly degraded in SFC^+^/Ext^+^/suc mice (*p < 0.05, one-way ANOVA, Bonferroni multiple comparison tests). **C**, **D**, **G** No differences were found for the phosphorylation levels of AKT Thr308, total P85 and PTEN. **H**–**N** Meg3-ex10 knockdown was induced 24 h after social fear acquisition in groups of SFC^−^/Ext^+^ and SFC^+^/EXT^+^ mice, and extinction training was performed 72 h thereafter. Protein and phosphorylation levels were measured in control and Meg3-ex10 knockdown animals at 90 min after social fear extinction training. **I** Phosphorylation levels of P85 were upregulated in SFC^+^/Ext^+^ control mice (*p < 0.05 vs. SFC^−^/Ext^+^ control, separate statistics: unpaired *t*-test). Meg3-ex10 knockdown increased baseline activity in SFC^−^/Ext^+^ knockdown mice by trend ((#)*p* = 0.06 vs. SFC^−^/Ext^+^ control, separate statistics: unpaired *t*-test). **J**–**N** Phosphorylation levels of AKT Ser473 and AKT Thr308, and total P85, AKT and PTEN levels were neither affected by social fear extinction nor by Meg3-ex10 knockdown. Data are presented as mean fold changes + SEM. Group sizes (left site; 3 h): *n* = 5–10. Group sizes (right site; 90 min): n(SFC^−^/Ext^+^ control) = 7–10; n(SFC^+^/Ext^+^ control) = 9–11; n(SFC^−^/Ext^+^ knockdown) = 9–10; n(SFC^+^/Ext^+^ knockdown) = 9–12.

At 90 min after the extinction training, no differences within the PI3K/AKT signaling pathway were observed between the groups (Figure S4). However, at 3 h after social fear extinction training, the PI3K/AKT signaling pathway was activated within the septum of SFC^+^/Ext^+^/unsuc (Fig. 4A–G) seen by increased phosphorylation of the P85 regulatory subunit of the PI3K by 1.7-fold (vs. SFC^−^/Ext^+^; Fig. 4B). Phosphorylation of the downstream kinase AKT at Ser473 was significantly increased in SFC^+^/Ext^+^/unsuc animals (Fig. 4E), whereas no differences were detected for the phosphorylation levels of AKT Thr308 (Fig. 4D). Total P85 and protein levels of PTEN, a negative regulator and a phosphatidyl-3,4,5-triphosphate phosphatase, were unchanged (Fig. 4C, G), while total AKT was significantly downregulated in SFC^+^/Ext^+^/suc compared with SFC^−^/Ext^+^ at 3 h after social fear extinction (Fig. 4F).

To understand how levels of Meg3-ex10 affect the activation of PI3K/AKT, we investigated the activation of the pathway in response to Meg3-ex10 knockdown. GapmeRs were microinfused bilaterally into the septum 24 h after acquisition. After 72 h of incubation and recovery time, mice were subjected to extinction training and sacrificed 90 min later (Fig. 4H–N). P85 phosphorylation levels were increased in SFC^+^/Ext^+^ control mice compared with SFC^−^/Ext^+^ control mice, indicating activation of PI3K at 90 min after social fear extinction training (Fig. 4I). However, a similar activation was not found in SFC^+^/Ext^+^ knockdown, when compared with SFC^−^/Ext^+^ knockdown mice. Interestingly, in SFC^−^/Ext^+^ animals Meg3-ex10 knockdown resulted in slightly increased P85 phosphorylation levels compared with SFC^−^/Ext^+^ control mice, and this increase in basal P85 phosphorylation after Meg3-ex10 knockdown might prevent further activation in the SFC^+^/Ext^+^ knockdown group after extinction training. No difference either in phosphorylation levels at AKT Thr308 and Ser473, or total P85, AKT, or PTEN were detected at 90 min after the extinction training (Fig. 4J–N).

### Extinction success and knockdown of Meg3-ex10 influence chromatin states

**Fig. 5:**
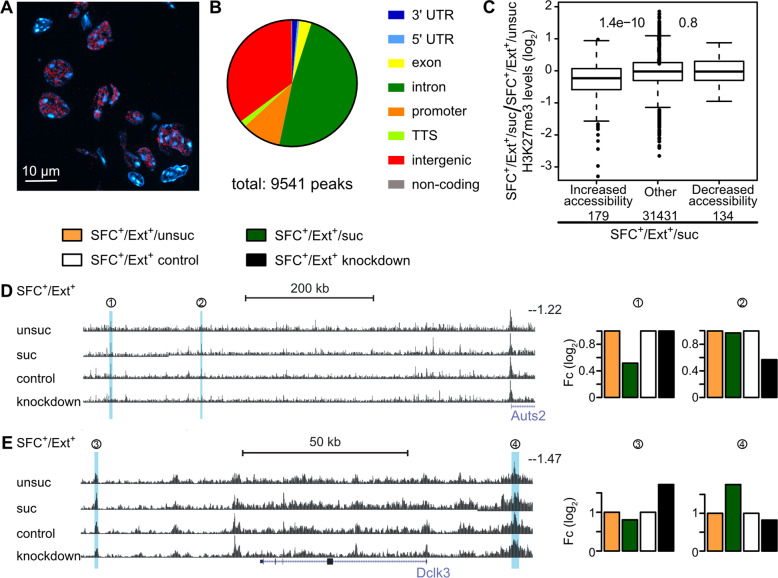
Extinction success and Meg3-ex10 knockdown alter chromatin states. **A** Septal brain slices from mice subjected to social fear extinction and sacrificed 90 min thereafter were used to visualize Meg-ex10 RNA localization by RNAscope. **B** Pie plot of the percentage of ATAC-seq peaks found in different regions of the genome in SFC^−^/Ext^+^, SFC^+^/Ext^+^/suc, and SFC^+^/Ext^+^/unsuc as well as SFC^+^ control and SFC^+^ Meg3-ex10 knockdown groups. Peaks are summarized into 3′ untranslated region (3′ UTR), 5′ untranslated region (5′ UTR), exons, introns, promoter transcription start sites (TSS), transcription termination sites (TTS), intergenic regions, and non-coding regions. **C** Correlation of ATAC-seq peaks and CUT&RUN H3K27me3 signals for SFC^+^/Ext^+^/suc vs. SFC^+^/Ext^+^/unsuc. The *y*-axis shows log_2_-fold change and numbers between the boxes represent *p*-values of Wilcoxon rank-sum test (vs. other). Numbers below the names on the *x*-axis indicate how many peaks meet the criteria (threshold fold change ≥ 33%, *p* < 0.05). The right panels show representative peaks around the Auts2 (**D**) and Dclk3 (**E**) loci, identified by ATAC-seq. Blue colored areas represent Uber_peaks (Auts2: (1)Uber_peak_135193, (2) Uber_peak_135189; Dclk3: (3) Uber_peak_177423, (4) Uber_peak_177395; for details see Supplementary Data 5), where significant differences between SFC^+^/Ext^+^/unsuc vs. SFC^+^/Ext^+^/suc and SFC^+^/Ext^+^ control vs. SFC^+^/Ext^+^ knockdown were detected. The middle and right panels represented fold changes (Fc) of all three replicates per group used for ATAC-seq. SFC^+^/Ext^+^/suc were normalized to SFC^+^/Ext^+^/unsuc, SFC^+^/Ext^+^ knockdown to SFC^+^/Ext^+^ control.

Using RNAscope we found a nuclear localization of Meg3-ex10 within the septum at 90 min after extinction training in all animals (Fig. 5A). This is in line with previous studies that additionally showed a capacity of Meg3 to alter chromatin states [36, 37]. Hence, we hypothesized that Meg3-ex10 affects chromatin states of its target genes after social fear extinction as well. To determine chromatin regions with altered accessibility that might correlate with Meg-ex10 levels, we performed an *Assay for Transposase-Accessible Chromatin with high-throughput sequencing* (ATAC-seq) [47] and *Cleavage Under Targets & Release Using Nuclease* (CUT&RUN) [49] for altered H3K27me3 modifications. Both methods allow for the identification of transcriptionally active genomic regions and thus, the identification of Meg3-ex10 target genes that are affected during extinction training. Septum samples from SFC^–^/Ext^+^, SFC^+^/Ext^+^/suc and SFC^+^/Ext^+^/unsuc were collected at 90 min after social fear extinction training and from SFC^+^ control and SFC^+^ Meg3-ex10 knockdown mice (microinfusion 24 h after social fear acquisition, extinction training 72 h later, behavioral data see Figure S5). To assess differential accessibility of loci, regions ─70 and +70 bp around the peak summit were called for the ATAC-seq data. We only considered regions with ≥ 5 reads per million reads in at least three samples. Using less stringent criteria (unadjusted *p* < 0.05), we identified 9 541 differentially expressed peaks (Fig. 5B). While most of the peaks were found in intronic and intergenic regions, approximately 11% were located in promoter regions and transcription start sites (Fig. 5B).

For CUT&RUN, we considered differential coverage of the broad H3K27me3 modification in regions -300 and +300 bp around the ATAC-seq peak summits. Since both methods identify chromatin states, we correlated the identified peaks from ATAC-seq and CUT&RUN (Supplementary Data 5). We found a significant correspondence between the changes in accessibility and H3K27me3 levels for the comparison between SFC^+^/Ext^+^/suc and SFC^+^/Ext^+^/unsuc samples, where regions with increased accessibility preferentially lost H3K27me3, consistent with its repressive nature (Fig. 5C).

We further assessed whether gene loci were similarly accessible in SFC^+^/Ext^+^/suc vs. SFC^+^/Ext^+^/unsuc, and SFC^+^ control vs. SFC^+^ knockdown mice (Supplementary Data 5). These comparisons were selected based on prominent differences in Meg3-ex10 levels found in these groups. Similar peaks were found in the intergenic region of the autism susceptibility gene2 (*Auts2*) and doublecortin-like kinase 3 (*Dclk3*) (GEO: GSE178210, Fig. 5D, E; Supplementary Data 5). *Auts2* was more accessible under conditions for low Meg3-ex10 levels, i.e., both in SFC^+^/Ext^+^/unsuc as well as in SFC^+^ Meg3-ex10 knockdown mice (intergenic region, see Supplementary Data 5: Uber_peak_135193, Uber_peak_135189). *Dclk3*, in contrast, was less accessible under low Meg3-ex10 conditions mice (intergenic region, see Supplementary Data 5: Uber_peak_177395, Uber_peak_177423).

We further investigated whether the alterations in chromatin accessibility result in transcriptomic changes. Hence, Auts2 and Dclk3 mRNA levels were first quantified both at 90 min as well as 3 h after extinction (Figure S6). Contrary to the more accessible chromatin indicated in the ATAC-seq data in mice with unsuccessful extinction and low Meg3-ex10 levels at the 90 min time point, Auts2 mRNA levels were significantly downregulated in SFC^+^/Ext^+^/unsuc (Figure S6A). At 3 h after extinction, however, Auts2 mRNA levels were restored (Figure S6B), hinting towards transcription dynamics at the *Auts2* locus. Consequently, we additionally investigated Auts2 expression levels at 5 h after extinction. Here significant decreased levels were detectable in SFC^−^/Ext^+^ mice (Figure S6C). For Dclk3 mRNA levels, no significant changes were detected, however, dynamics in transcription are indicated within the time frame of 90 min to 5 h after extinction training (Figure S6E–G). Auts2 and Dclk3 mRNA were not altered in Meg3-ex10 knockdown and control mice 90 min after extinction (Figure S6D, H), which might be a matter of identifying the specific time point as indicated by the strong dynamics during naturally occurring extinction mentioned before.

## Discussion

Using a murine model of social fear conditioning, we provide an in-depth transcriptomic characterization of social fear acquisition and its extinction, and first insights into chromatin alterations within the septum that are associated with the extinction process. We also revealed local RNA changes on an isoform level, supporting recent findings on the significance of splicing events in behavioral regulation in health and disease [59–61]. Specifically, we identified isoforms of the lncRNA Meg3 (Meg3-ex10), which were negatively affected by social fear conditioning, but restored after successful social fear extinction, with a positive correlation of Meg3-ex10 levels with the success of social fear extinction. Although a robust functional link between the knockdown of Meg3-ex10 in the septum and impaired social fear extinction has not been detected, we conclude an association between septal Meg3-ex10 levels and the success of social fear extinction. The regulation of Meg3-ex10 in social fear extinction was accompanied by an increased baseline activity of the PI3K/AKT signaling pathway in Meg3-ex10 knockdown mice and shifted activation in mice with unsuccessful extinction. Based on the nuclear localization of Meg3-ex10 within the septum after social fear extinction, we further identified chromatin regions, which were affected in their accessibility and H3K27me3 state in dependence of Meg3-ex10 levels, using ATAC-seq and CUT&RUN. In this way, we identified *Auts2* and *Dclk3* as potential targets of Meg3-ex10. Based on our results, we propose a working model, in which the lncRNA Meg3-ex10 is actively regulated by social fear and the success of fear extinction in the septum of the mouse brain. Meg3-ex10 regulation leads subsequently to altered signaling of the PI3K/AKT signaling pathway and chromatin states. Such Meg3-ex10-mediated changes may further influence plasticity events, which are necessary for the extinction or social fear memory.

The mouse model of SFC enables us to investigate underlying neurobiochemical mechanisms of social fear and social avoidance - the major symptoms of SAD [14, 62]. The difficult diagnosis of SAD usually occurs after social phobia has already been manifested. Therefore, from a translational research perspective, we were particularly interested in transcriptional changes accompanying social fear extinction training, which mimics exposure therapy applied in humans with SAD [7, 63]. Importantly, not only resilience and susceptibility for SAD, but also rates of therapeutic responsiveness and relapse show substantial inter-individual differences [8, 63–65]. However, in previous SFC studies, the naturally occurring variance in the outcome of extinction training performed on day 2 of the SFC paradigm has largely been ignored [12, 14, 66]. Here we show that, indeed, the success of social fear extinction strongly determined the septal expression of the Meg-ex10 isoforms (Fig. 1), and the maintenance of social fear was accompanied by the maintenance of reduced Meg3-ex10 levels. (Fig. 2A, Fig. 1D). This suggests that septal Meg3-ex10 levels are an indicator for successful or unsuccessful fear extinction and, consequently, are inversely correlated to social fear, i.e., higher Meg3-ex10 expression correlates with reduced social fear expression during extinction. In this line, it is well established that fear acquisition and extinction involve distinct processes of learning and memory [67–69]. Therefore, the opposing regulation of Meg3-ex10 within the septum after social fear acquisition and after successful fear extinction might be based on these different types of learning as also shown for other molecules [70–73]. Interestingly, septal Meg3-ex10 expression was found to be strictly dependent on social fear extinction learning (Fig. 2B), but independent of social interactions per se (Fig. 2C). However, with our experimental setup we cannot decipher, whether the social component of extinction learning, or the learning process in general including non-social learning, are mandatory for Meg3-ex10 regulation. Considering the fact that the SFC model is based on operant fear conditioning principles, we could not include contextual or cued fear conditioning as an appropriate control. However, a recently published paradigm based on non-social instrumental fear conditioning [74] might be applied as control for future studies to examine social-specificity vs. broader relevance in instrumental fear conditioning of the targets identified in our study.

So far, Meg3 isoforms have only rarely been characterized in a behavioral context, and previous studies either focused on the common Meg3-short isoform or did not specify them [30, 31, 75]. By specifying the expression of Meg3 isoforms we could identify the exclusive regulation of Meg3-ex10, as neither total Meg3 nor Meg3-short levels were found to be altered after successful social fear extinction (Figure S2C–F), also confirmed by gene- and transcript-based analyses of RNA-seq data. Moreover, Meg3-ex10 levels were specifically regulated within the septum (Figure S2G–J), an important relay station for emotional processing [76] and a hot spot for regulating various socio-emotional behaviors [16, 77, 78], including social fear behavior [12, 14]. The local knockdown experiments, however, could not fully confirm our initial hypothesis that decreased Meg3-ex10 levels within the septum contribute to impaired social fear extinction (Fig. 3B, G). Although social fear conditioning (24 h timepoint) and fear extinction regulate Meg3-ex10 expression, only knockdown of septal Meg3-ex10 prior to, but not after social fear acquisition and 72 h prior to extinction, delayed extinction outcomes. In this context, potential compensatory mechanisms that take over Meg3-ex10 functions during the 72-hour post-knockdown need to be considered, since behavioral experiments were conducted 72 h after surgery for Meg3-ex10 knockdown. Moreover, the manipulation of Meg3-ex10 as a lncRNA with regulatory functions might not result in on/off-effects in behavior. As we could only find an effect of Meg3-ex10 manipulation, when performed prior to social fear acquisition, future studies should also address the possibility that pre-existing differences in Meg3-ex10 expression may mediate acquisition or post-acquisition processes, which subsequently affect the success of social fear extinction. Additionally, various downstream targets of Meg3-ex10 are likely to play a role in mediating Meg3-ex10 effects and, consequently, their identification and direct manipulation may result in more prominent behavioral alterations. Although, according to our hypothesis, overexpression of Meg3-ex10 might facilitate fear extinction, it is currently impossible to perform these experiments due to the length of the transcript (~12 kb) and available methodology. Furthermore, Meg3-ex10 is only one lncRNA out of many components of a complex epigenetic network [24] and thus, regulation of social behaviors may require fine-tuning of such a multifaceted network on many different levels.

The regulation of Meg3 during learning and memory processes is in agreement with previous reports on its role in plasticity processes [30, 31], although we could not identify any effects on extinction and memory consolidation after Meg3-ex10 knockdown in our setup with social context (Fig. 3C).

Stating the above, we further focused on two well studied potential downstream mechanisms of Meg3, the PI3K/AKT signaling pathway [30, 31, 38] and modifications at a chromatin level [25, 28, 79]. Although the regulatory functions of Meg3-ex10 are well known, there is little knowledge regarding the temporal dynamics of its effects on PI3K/AKT signaling. Hence, we investigated phosphorylation status and protein levels of components 90 min and 3 h after extinction training, when a peak of Meg3-ex10 differences was observed. The PI3K/AKT phosphorylation states after social fear extinction (Fig. 4A–G, S4) hint towards a delayed activation of the PI3K/AKT signaling pathway in animals with unsuccessful extinction, in which full activation of AKT by additional pAKT Thr308 is likely in progress [80]. However, there is also evidence that partial activation of AKT at Ser473 is sufficient to mediate plasticity and memory consolidation [81]. Contrary to our expectations, no activation was found in mice with successful extinction, even though learning processes and hence, plasticity events were ongoing (Fig. 4B–G, S4). Supported by decreased AKT protein levels after 3 h of extinction (Fig. 4F), the PI3K/AKT signaling pathway might become activated earlier, since learning occurs already with the first approaches in mice with successful extinction [82]. A direct link between Meg3-ex10 and the PI3K/AKT signaling pathway could also be established in the Meg3-ex10 knockdown experiments. Hyperactivation of PI3K was found in Meg3-ex10 knockdown animals, similar to in vitro studies for long-term potentiation using cortical neurons with Meg3 knockdown [31]. This hyperactivation likely prevents further activation, which was observed in conditioned control mice (Fig. 4I). Interestingly, this activation has already been detected at the 90-min timepoint. As we also have to consider the 72 h delay in the knockdown experiments, a direct comparison of data shown in Fig. 4A–G and Figure S4 is not useful. This could also explain the discrepancy of delayed activation and hyperactivation seen in conditioned animals with unsuccessful extinction and an acute Meg3-ex10 regulation versus conditioned knockdown animals with long-term Meg3-ex10 regulation. Therefore, a more detailed investigation of the extent, timing, and duration of PI3K/AKT signaling is needed for further dissection of signaling mechanisms downstream of Meg3-ex10, which are regulated by extinction success. Moreover, the differentiation in nuclear and cytoplasmatic PI3K/AKT signaling should be considered; so far, we have identified only a nuclear localization of Meg3-ex10 within the septum (Fig. 5A). Nuclear PI3K/AKT signaling has been described to regulate ribosome biogenesis, cell survival and transcriptional processes [83, 84].

At chromatin level, we found two interesting loci, *Auts2* and *Dclk3*, which are similarly up- and downregulated in their accessibility, respectively, in the groups with low Meg3-ex10 levels (i.e., unsuccessful extinction and Meg3-ex10 knockdown). *Auts2* has been linked to many psychiatric disorders, including autism spectrum disorders and schizophrenia, and animal studies demonstrated that AUTS2 regulates emotional control, cognitive memory and social communication in mice [85–87]. Within the cell nucleus, it acts as transcriptional activator by interacting with the PRC1 and the histone acetyltransferase P300 [88], whereas cytoplasmatic AUTS2 plays a role in cytoskeletal rearrangements [89]. The function of DCLK3 is so far unknown. However, it has been linked to neuroprotective properties in Huntington’s disease [90–94]. Correlating chromatin alterations to transcriptional dynamics is challenging considering the fact that temporal kinetics of both processes depend on multiple factors at multiple levels, where ATAC-seq peaks and transcript levels from RNA-seq represent a snapshot of this complex dynamics. This ambiguity is evident in our validation of the two candidate genes *Auts2* and *Dclk3* at various time points after extinction training. Although we chose time points relevant to Meg3-ex10 regulation and the PI3K/Akt signaling pathway we did not observe a positive correlation between the chromatin alterations observed and the transcript levels. For example, Auts2 levels seem to increase from 90 min to 5 h in the unsuccessful extinction group, reaching comparable levels to successful extinction group, but significantly higher than unconditioned mice. For Dclk3 too, mRNA levels indicate the possibility that chromatin states might have been established at earlier time points that still persist at 90 min after extinction. However, these conclusions are speculative and need to be further investigated. Our validation experiments were also restricted to available samples, i.e., from Meg3-ex10 knockdown at 90 min, and ethical concerns in adding more cohorts in this study. This will require future experiments including additional time points in Meg3-ex10 knockdown after acquisition and extinction.

In summary, the study is first of its kind exploring the transcriptome and first chromatin level modifications accompanying social fear and its extinction. These results lead to interesting questions for further studies including the identification of downstream targets of Meg3-ex10 in more detail, whose direct manipulation might result in more prominent effects on social fear extinction and memory consolidation.

## Supplementary information

Supplementary Figure 1

Supplementary Figure 2

Supplementary Figure 3

Supplementary Figure 4

Supplementary Figure 5

Supplementary Figure 6

Supplementary Figures Legends

Supplementary Materials

Supplementary Tables

Supplementary Data 1_Gene_based_SFC_SFCp combined

Supplementary Data 2_Gene_based_SFC_SFCp extinction success

Supplementary Data 3_Transcript_based_SFC_SFCp combined

Supplementary Data 4_Transcript_based_SFC_SFCp extinction success

Supplementary Data 5_mergedATACandCR.joint

## Data Availability

All the RNA-seq, ATAC-seq, and CUT&RUN data have been deposited in the GEO database under ID code GSE178210.
