# Supplementary figures and images for: Transcriptome and chromatin alterations in social fear indicate association of MEG3 with successful extinction of fear
Source: Mol Psychiatry. 2022 Mar 25;27(10):4064–76. doi: 10.1038/s41380-022-01481-2 (PMC9718683; doi:10.1038/s41380-022-01481-2)

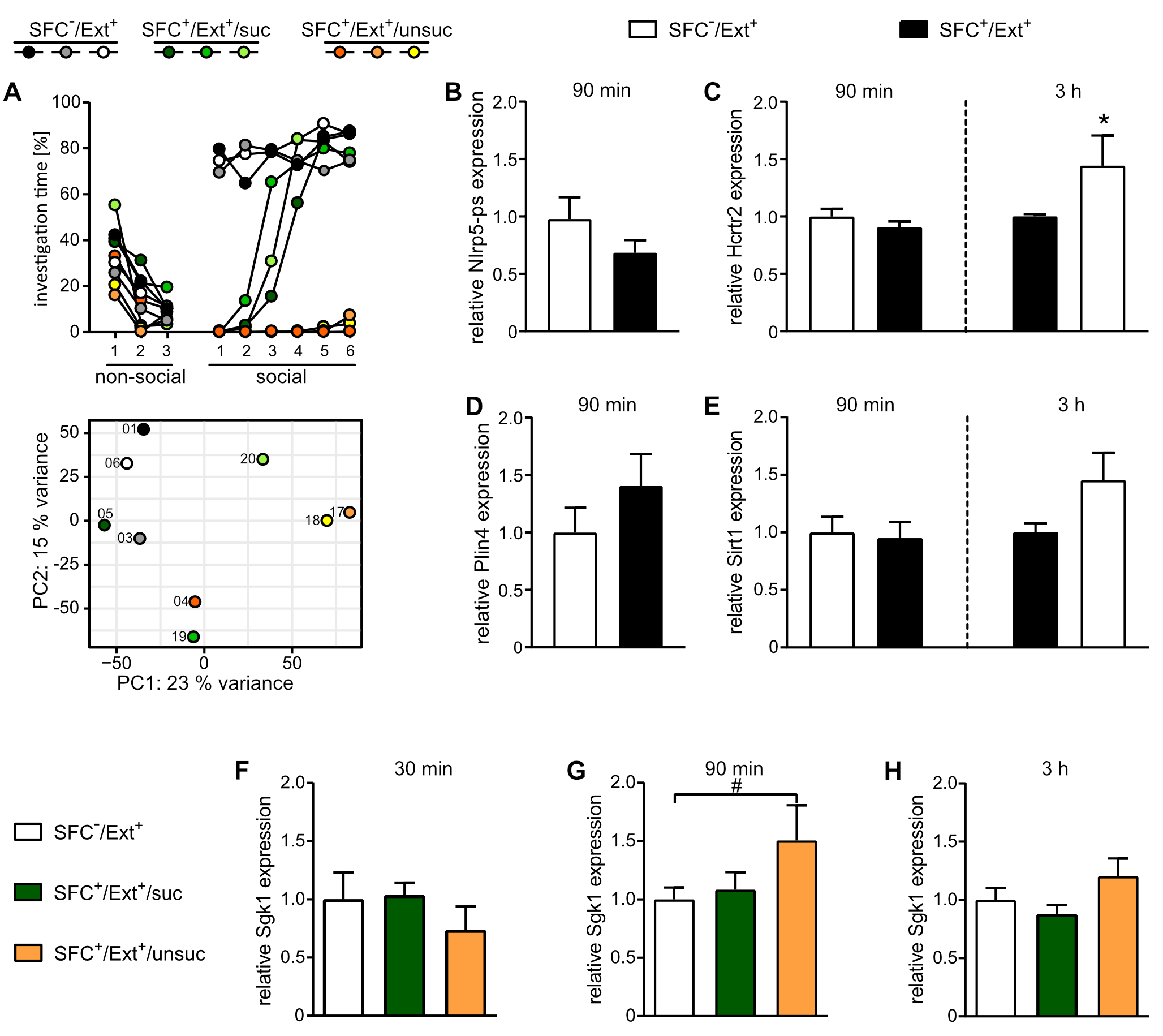

Supplement: Supplementary file 1 — Supplementary Figure 1 [file 41380_2022_1481_MOESM1_ESM.tif]

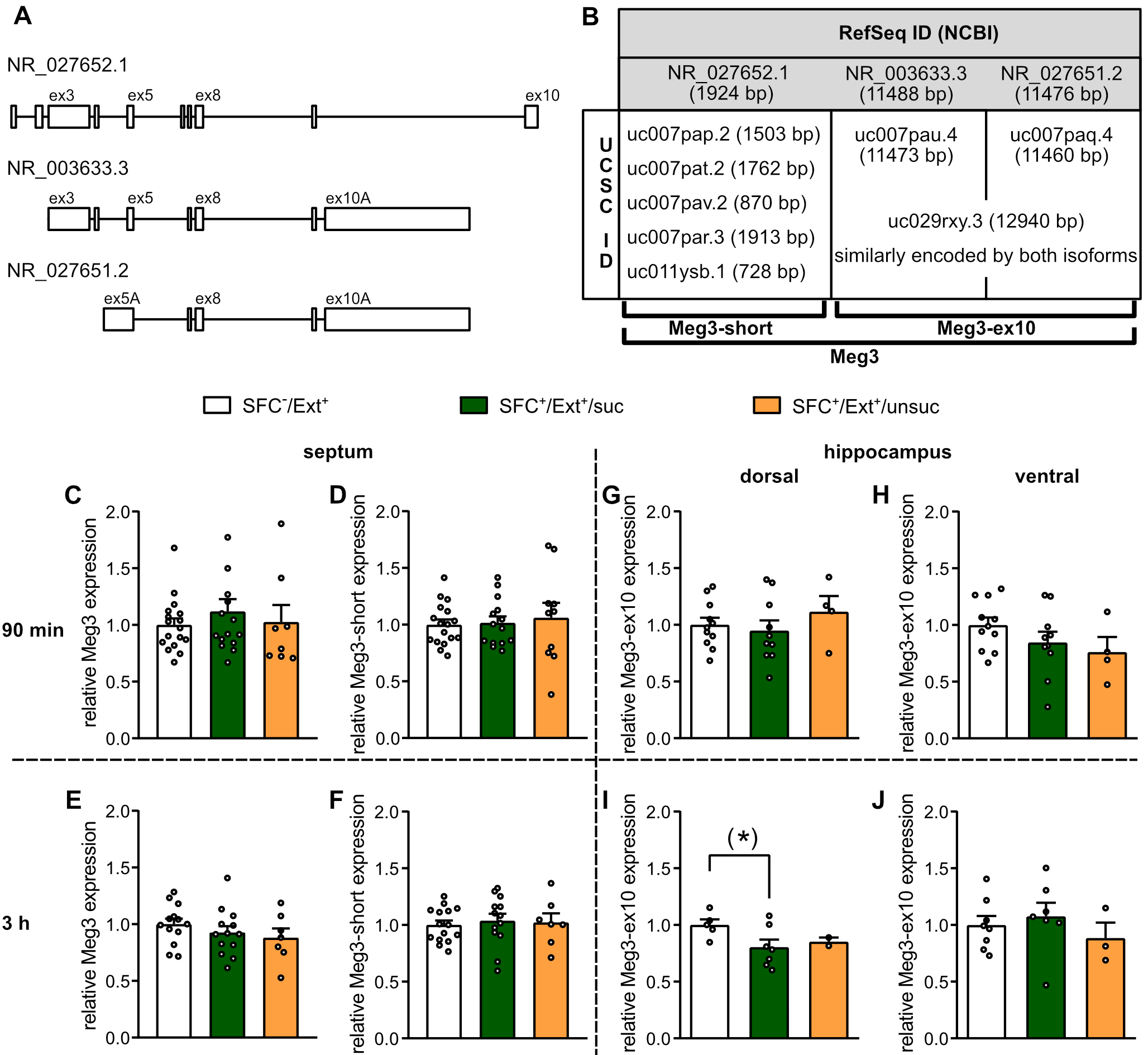

Supplement: Supplementary file 2 — Supplementary Figure 2 [file 41380_2022_1481_MOESM2_ESM.tif]

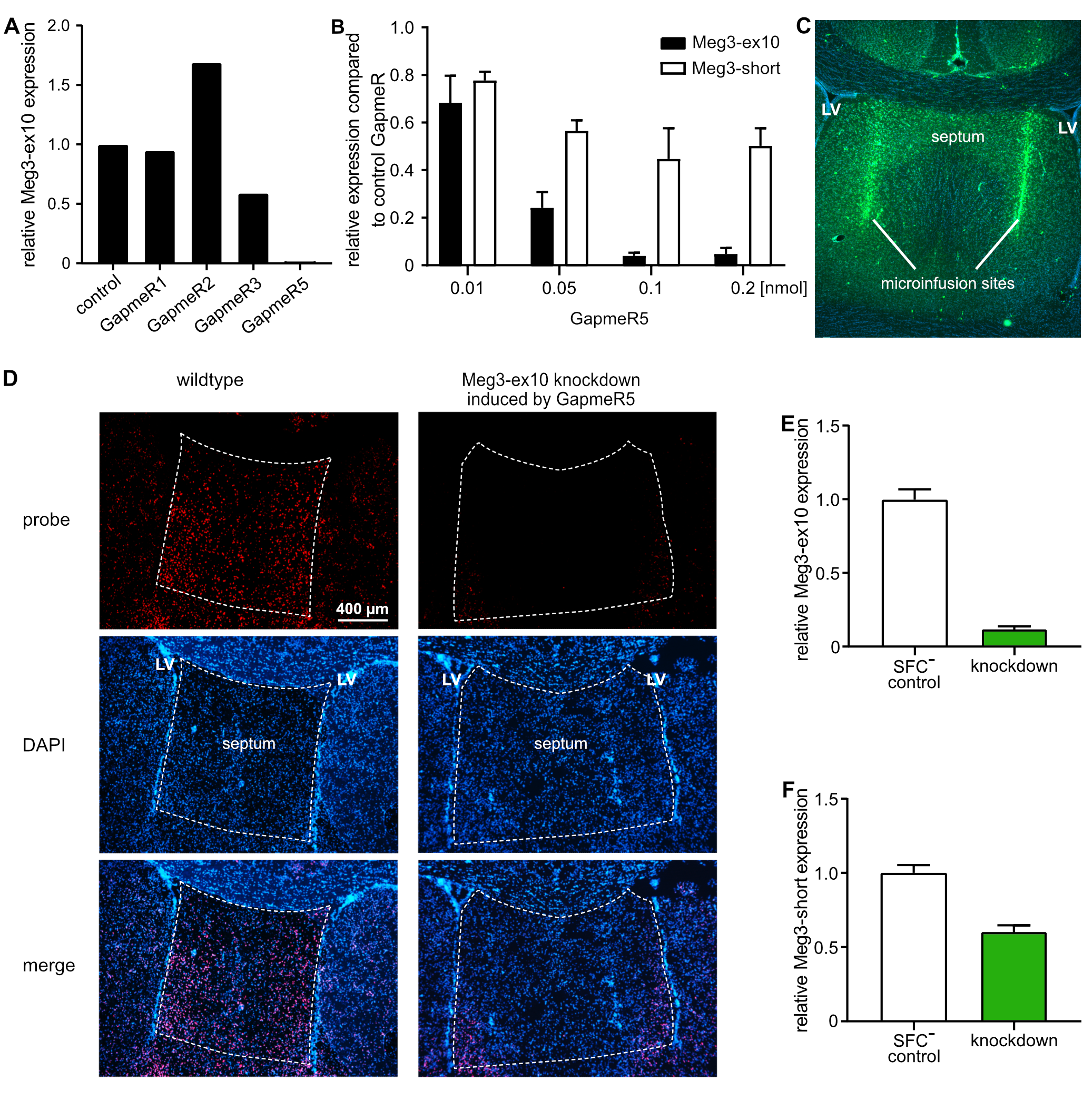

Supplement: Supplementary file 3 — Supplementary Figure 3 [file 41380_2022_1481_MOESM3_ESM.tif]

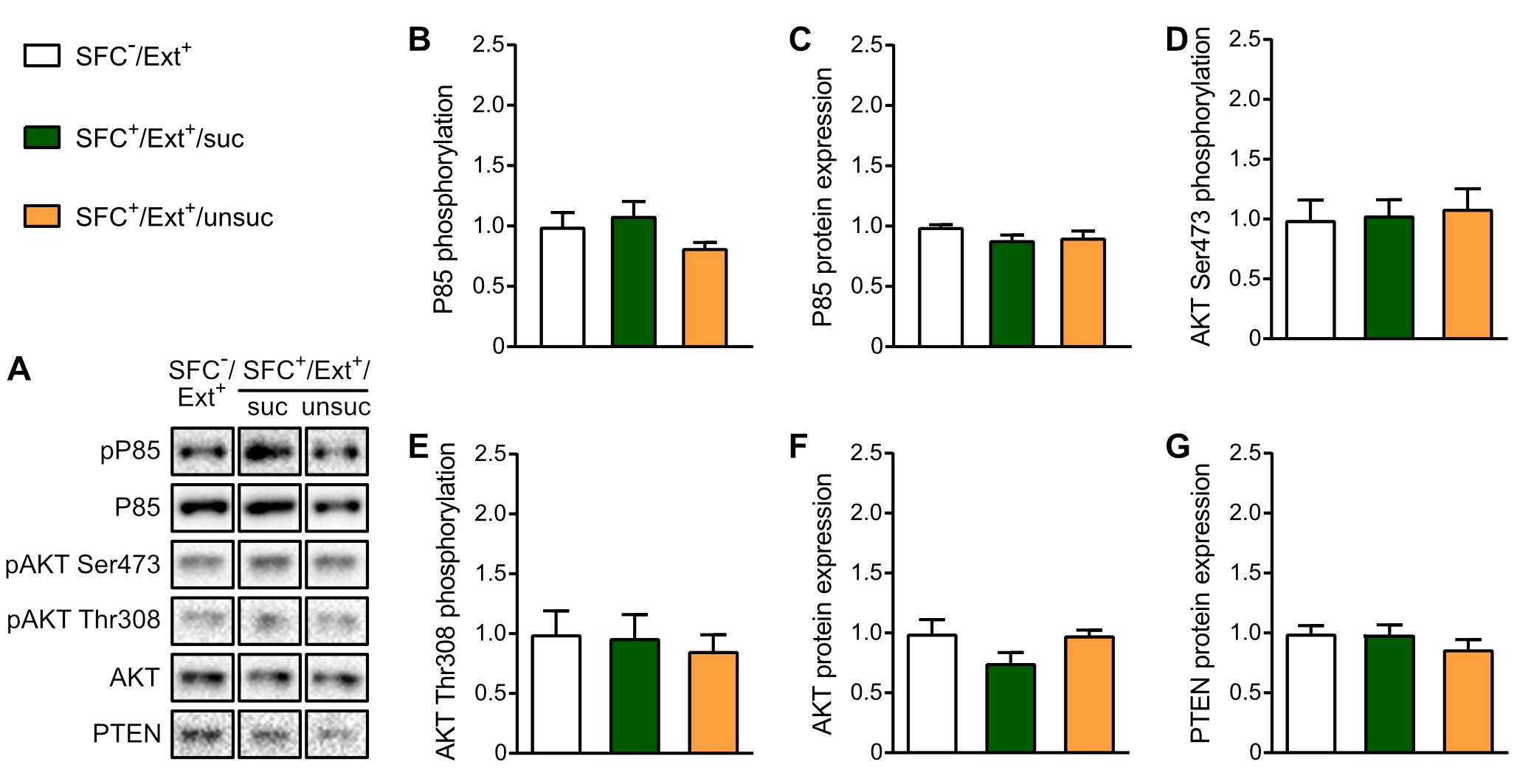

Supplement: Supplementary file 4 — Supplementary Figure 4 [file 41380_2022_1481_MOESM4_ESM.tif]

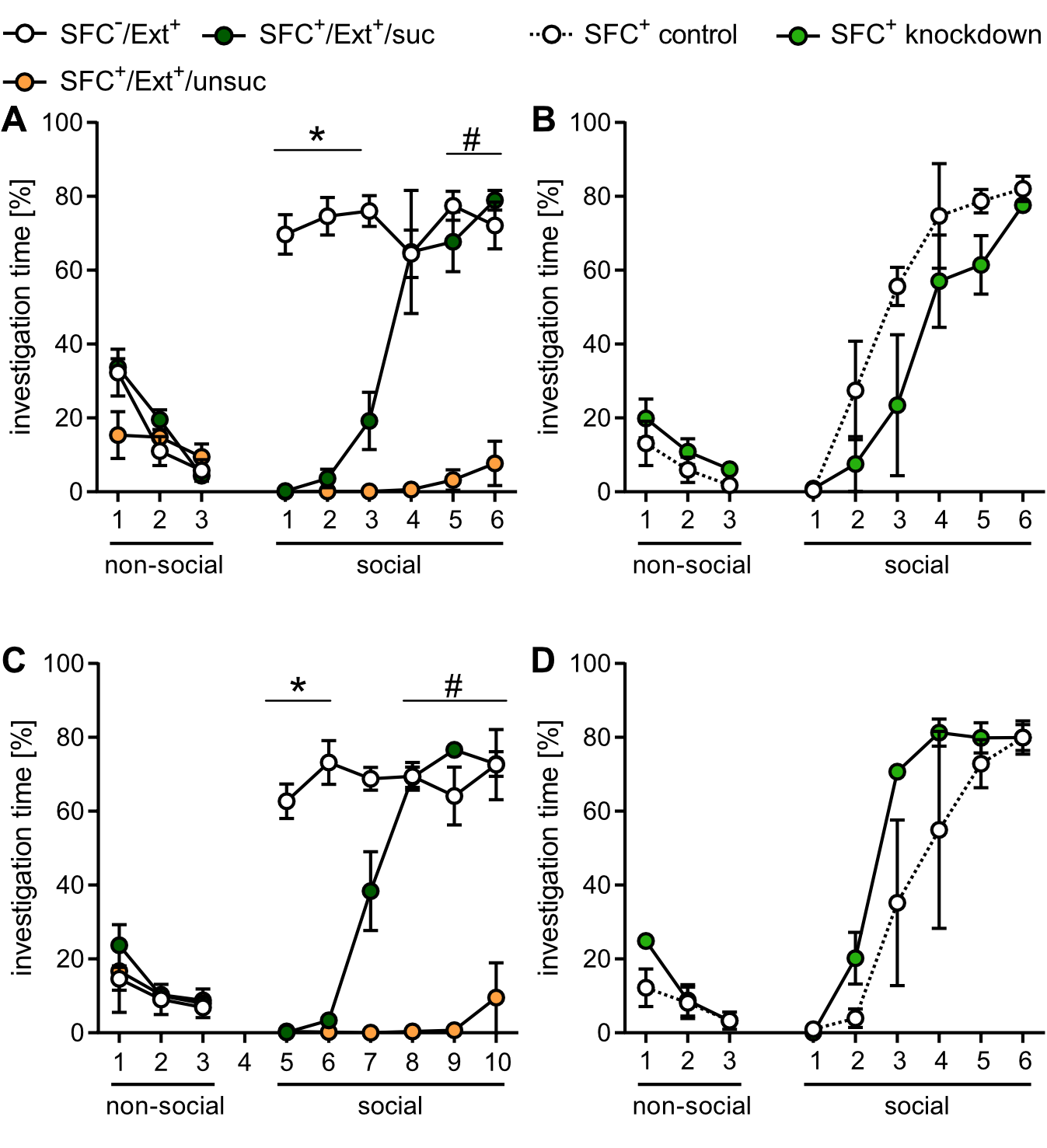

Supplement: Supplementary file 5 — Supplementary Figure 5 [file 41380_2022_1481_MOESM5_ESM.tif]

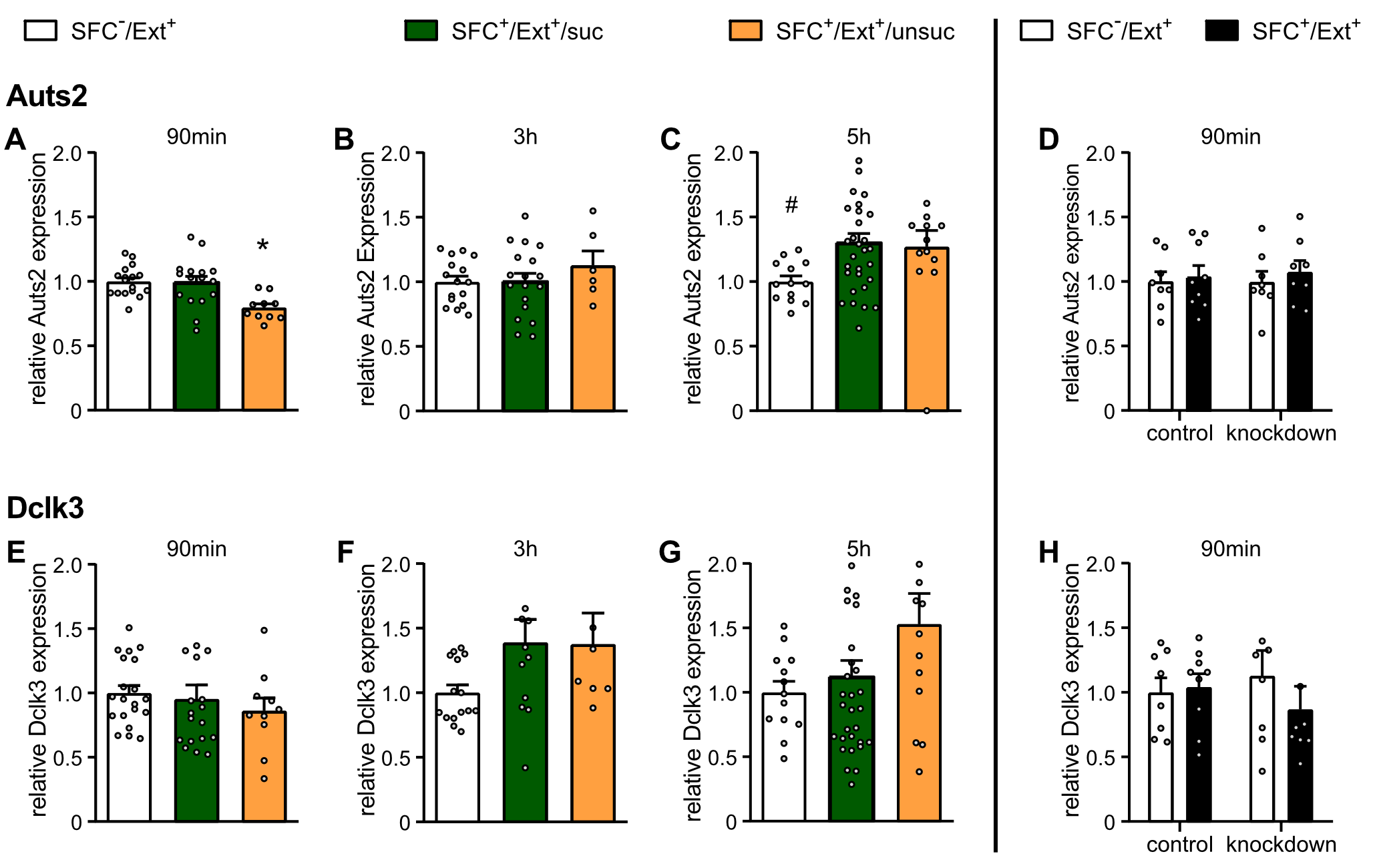

Supplement: Supplementary file 6 — Supplementary Figure 6 [file 41380_2022_1481_MOESM6_ESM.tif]
