# Supplementary material for: Transcriptome and chromatin alterations in social fear indicate association of MEG3 with successful extinction of fear
Source: Mol Psychiatry. 2022 Mar 25;27(10):4064–76. doi: 10.1038/s41380-022-01481-2 (PMC9718683; doi:10.1038/s41380-022-01481-2)
Supplement: Supplementary file 7 — Supplementary Figures Legends [file 41380_2022_1481_MOESM7_ESM.docx]

**SUPPLEMENTARY FIGURES TITLES AND LEGENDS**

**Supplementary Figure 1 Validation of RNA-seq data confirmed dynamic regulation of coding and non-coding RNAs.**

1. Upper panel: Individual investigation time of three non-social (empty cages) and six social (conspecifics) stimuli during social fear extinction training of mice with successful (SFC^+^/Ext^+^/suc; green colors) and unsuccessful (SFC^+^/Ext^+^/unsuc, yellow/orange colors) fear extinction and their unconditioned controls (SFC^-^/Ext^+^, black and white colors) that were used for total RNA-seq. Lower panel: Principle component analysis of RNA-seq data analyzed with StringTie/DESeq2 on a transcript-based level.

(B-E) Relative expression of the non-coding RNA Nlrp5-ps and coding RNAs Hcrtr2, Plin4 and Sirt1. RNA levels of SFC^-^/Ext^+^ and SFC^+^/Ext^+^ mice were regulated in the way observed in the RNA-seq data, however levels at 90 min after social fear extinction training have not reached statistical significance. (C) Indeed, Hcrtr2 mRNA levels were significantly upregulated in SFC^+^/Ext^+^ mice at 3 h after the social fear extinction training (*p = 0.01, Mann-Whitney test). Group sizes: 90 min: n(SFC^-^/Ext^+^) = 15-18, n(SFC^+^/Ext^+^) = 12-18; 3 h: n(SFC^-^/Ext^+^) = 7-8, n(SFC^+^/Ext^+^) = 12).

(F-H) Sgk1, an immediate early gene and another candidate from RNA-Seq, was dynamically regulated after social fear extinction. Assessment of RNA levels after 30 min, 90 min and 3 h showed increased Sgk1 levels in SFC^+^/Ext^+^/unsuc at 90 min after social fear extinction (#p = 0.0507 vs. SFC^-^/Ext^+^, separate statistics: unpaired t-test). Group sizes (Sgk1 mRNA levels): 30 min: n(SFC^-^/Ext^+^) = 7, n(SFC^+^/Ext^+^/suc) = 5, n(SFC^+^/Ext^+^/unsuc) = 2; 90 min: n(SFC^-^/Ext^+^) = 20, n(SFC^+^/Ext^+^/suc) = 12, n(SFC^+^/Ext^+^/unsuc) = 6; 3 h: n(SFC^-^/Ext^+^) = 10, n(SFC^+^/Ext^+^/suc) = 10, n(SFC^+^/Ext^+^/unsuc) = 4.

Data are presented as mean fold changes + SEM compared with respective SFC^-^.

**Supplementary Figure 2 Meg3 is isoform- and region-specifically regulated after social fear extinction.**

(A-B) Overview of murine Meg3 isoforms. (A) Three mouse Meg3 isoforms are annotated in RefSeq, the sequence database of NCBI. NR_027652.1 contains 10 exons. NR_003633.3 and NR_027651.2 contain a large alternative exon 10 (10A). NR_027651.2 has additionally an alternative exon 5 (5A) (adapted from ^75^). (B) Eight Meg3 isoforms were found in the RNA-seq aligned to mm10 and compared with the database of the UCSC Genome Browser. Isoforms annotated in RefSeq include the sequences of the UCSC annotated isoforms.

(C-F) Neither Meg3 on a gene-based level (all isoforms) nor the Meg3-short isoform were differentially regulated within the septum in SFC^-^/SFC^+^, SFC^+^/Ext^+^/suc and SFC^+^/Ext^+^/unsuc at 90 min (n=8-13) and 3 h after the social fear extinction training. Group sizes: n=7-15.

(G-J) Meg3-ex10 was not regulated within the dorsal nor the ventral hippocampus at 90 min or 3 h after social fear extinction. After 3 h, dorsohippocampal Meg3-ex10 was by trend downregulated in SFC^+^/Ext^+^/suc ((*)p = 0.0573 SFC^+^/Ext^+^/suc vs. SFC^-^/Ext^+^, separate statistics: unpaired t-test). Group sizes: 90 min: n(SFC^-^/Ext^+^) = 11, n(SFC^+^/Ext^+/^suc) =11, n(SFC^+^/Ext^+^/unsuc) = 4; 3 h: n(SFC^-^/Ext^+^) = 5-8, n(SFC^+^/Ext^+^/suc) = 7, n(SFC^+^/Ext^+^/unsuc) = 2-3.

Data represent mean fold changes + SEM compared with respective SFC^-/^Ext^+^ group. Circles: individual data points.

**Supplementary Figure 3 Establishment of antisense LNA GapmeRs for in vivo Meg3-ex10 knockdown.**

(A) Different antisense LNA GapmeRs (0.2 nmol per animal) for Meg3-ex10 knockdown were tested with GapmeR5 being the most efficient one. Meg3-ex1 levels were determined after 72h incubation time.

(B) Dose-response curves revealed 0.1 nmol of GapmeR5 per animal as the most efficient concentration for in vivo Meg3-ex10 knockdown experiments within the septum. Meg3-short levels were minorly reduced and similarly expressed for all tested GapmeR5 concentrations (n = 2). Meg3-ex1 levels were determined after 72h incubation time. Data represent mean fold change + SEM normalized to corresponding control group.

(C) FAM-labeling of GapmeR5 (green) and (D) RNAscope for Meg3-ex10 (red) confirmed specific distribution of GapmeRs and Meg3-ex10 knockdown specifically within the mouse brain septum 72 h after microfinfusions.

(E-F) Relative Meg3-ex10 and Meg3-short expression levels of Meg3-ex10 knockdown mice at 90 min after extinction training. 0.1 nmol GapmeR5 or control GapmeR were microinfused at 24 h after social fear acquisition. Social fear extinction was performed 72 h later and animals were sacrificed 90 min after extinction training. Group sizes: n(SFC^-^ control) = 9, n(knockdown) = 17. LV: lateral ventricle, blue = DAPI, probe = specific RNAscope probe for Meg3-ex10.

**Supplementary Figure 4 The PI3K/AKT signaling pathway is not differentially activated in unconditioned and conditioned mice at 90 min after extinction training.**

1. Protein and phosphorylation levels were measured at 90 min after social fear extinction training in unconditioned mice (SFC^-^/Ext^+^), and in conditioned mice with successful (SFC^+^/Ext^+^/suc) and unsuccessful (SFC^+^/Ext^+^/unsuc) social fear extinction.

(B-G) No differences were found for the phosphorylation levels of P85 (pP85), AKT Ser473 and AKT Thr308. Total protein of P85, AKT and PTEN were similar in SFC^-^/Ext^+^, SFC^+^/Ext^+^/suc and SFC^+^/Ext^+^/unsuc.

Data are presented as mean fold changes + SEM compared with respective SFC^-^. Group sizes: 90 min: n = 7-13.

**Supplementary Figure 5 Social fear extinction behavior of samples used for ATAC-seq and CUT&RUN.**

Investigation time of three non-social (empty cages) and six social (conspecifics) stimuli during social fear extinction training of (A, C) mice with successful (SFC^+^/Ext^+^/suc) and unsuccessful (SFC^+^/Ext^+^/unsuc) fear extinction and their unconditioned controls (SFC^-^/Ext^+^) (*p < 0.05 SFC^+^/Ext^-^ vs. other; #p < 0.05 SFC^+^/Ext^+^/unsuc vs. other) and of (B,D) conditioned Meg3-ex10 knockdown and control mice. Meg3-ex10 knockdown was induced at 24 h after social fear acquisition and extinction training was performed 72 h later. Two-way ANOVA, Bonferroni multiple comparison tests.

Samples from A and B were used for ATAC-seq, samples from C and D for CUT&RUN for H3K27me3. Data represent mean investigation time ± SEM. Group sizes: n = 3 per group.

**Supplementary Figure 6 Validating ATAC-seq data by quantifying Auts2 and Dclk3 mRNA levels.**

(A-C) Relative expression of Auts2 mRNA in SFC^-^/Ext^+^, SFC^+^/Ext^+^/suc and SFC^+^/Ext^+^/unsuc mice 90 min, 3h and 5h after social fear extinction. At 90min, Auts2 mRNA levels were significantly decreased in SFC^+^/Ext^+^/unsuc mice (*p < 0.005 SFC^+^/Ext^+^/unsuc vs. other). Levels at 3h after social fear extinction training have not reached statistical significance, whereas at 5h, SFC^-^/Ext^+^ mice have significantly decreased levels compared to conditioned groups (#p < 0.03). Group sizes: 90 min: n(SFC^-^/Ext^+^) = 17, n(SFC^+^/Ext^+^/suc) = 16, n(SFC^+^/Ext^+^/unsuc) = 10; 3 h: n(SFC^-^/Ext^+^) = 16, n(SFC^+^/Ext^+^/suc) = 18, n(SFC^+^/Ext^+^/unsuc) = 6; 5h: n(SFC^-^/Ext^+^) = 13, n(SFC^+^/Ext^+^/suc) = 31, n(SFC^+^/Ext^+^/unsuc) = 13.

1. Meg3-ex10 knockdown was induced 24 h after acquisition and 72 h prior to social fear extinction. Animals were sacrificed 90min after social fear extinction. Auts2 mRNA levels were similar in all treatment groups. Group sizes: n(SFC^-^) = 8 per group, n(SFC^+^) = 8-9 per group.

(E-F) Relative expression of Dclk3 mRNA in SFC^-^/Ext^+^, SFC^+^/Ext^+^/suc and SFC^+^/Ext^+^/unsuc mice 90 min, 3h and 5h after social fear extinction. However, Dclk3 mRNA levels have not reached statistical significance at any investigated time point after social fear extinction training. Group sizes: 90 min: n(SFC^-^/Ext^+^) = 20, n(SFC^+^/Ext^+^/suc) = 18, n(SFC^+^/Ext^+^/unsuc) = 10; 3 h: n(SFC^-^/Ext^+^) = 15, n(SFC^+^/Ext^+^/suc) = 12, n(SFC^+^/Ext^+^/unsuc) = 7; 5h: n(SFC^-^/Ext^+^) = 13, n(SFC^+^/Ext^+^/suc) = 31, n(SFC^+^/Ext^+^/unsuc) = 13.

1. No differences in Dclk3 mRNA levels were detected for within Meg3-ex10 knockdown and control samples. Group sizes: n(SFC^-^) = 8 per group, n(SFC^+^) = 8-9 per group.

Data presented as mean fold change + SEM vs. SFC^-^/Ext^+^ or vs SFC^-^/Ext^+^ control. Circles: single data points.
