# Supplementary material for: Transcriptome and chromatin alterations in social fear indicate association of MEG3 with successful extinction of fear
Source: Mol Psychiatry. 2022 Mar 25;27(10):4064–76. doi: 10.1038/s41380-022-01481-2 (PMC9718683; doi:10.1038/s41380-022-01481-2)
Supplement: Supplementary file 8 — Supplementary Materials [file 41380_2022_1481_MOESM8_ESM.docx]

**SUPPLEMENTARY MATERIALS**

**Microinfusion of antisense LNA GapmeR:**

Mice were anaesthetized with isoflurane and placed in a stereotactic frame. The skull was exposed to antiseptic conditions and a small craniotomy was made with a thin drill over the septum (from Bregma +0.3 mm anteroposterior, ±0.5 mm mediolateral). Antisense LNA GapmeRs (custom designed, 3’-FAM-labeled, Qiagen, Hilden, Germany, Supplementary table 1) were bilaterally microinfused using a 5 µl calibrated micropipette (VWR, Darmstadt, Germany, inner diameter of 0.3 mm), which was pulled to create a long narrow shank. In total, 280 nl per animal (4x 70nl) were infused slowly by pressure infusion into the lateral septum. Two different dorsoventral positions were microinfused per hemisphere to guarantee the distribution of the antisense LNA GapmeRs exclusively, but within the total septum. Therefore, after the infusion at the first position (from Bregma +0.3 mm anteroposterior, ±0.5 mm mediolateral and –3.4 mm dorsoventral axis), the micropipette was kept in place for 30 s to ensure adequate diffusion. At position 2 (from Bregma +0.3 mm anteroposterior, ±0.5 mm mediolateral, and –3.0 mm dorsoventral axis), diffusion time was increased to 5 min to ensure that no antisense LNA GapmeRs are pulled to other regions while removing the micropipette. The wound was sutured with sterile nylon material

**ATAC-seq:**

Nuclear extraction buffer: 10mM Tris, 10mM NaCl, 3mM MgCl_2_, 0.1% Igepal, 0.1% Tween, protease inhibitor cocktail10mM Tris, 10mM NaCl, 3mM MgCl_2_, 0.1% Igepal, 0.1% Tween, protease inhibitor cocktail

**uliCUT&RUN:**

Nuclear extraction buffer: 20mM HEPES-KOH pH 7.9, 10mM KCl, 0.5mM Spermidine, 0.1% TritonX-100, 20% glycerol, protease inhibitor).
