# Supplementary material for: Transcriptome and chromatin alterations in social fear indicate association of MEG3 with successful extinction of fear
Source: Mol Psychiatry. 2022 Mar 25;27(10):4064–76. doi: 10.1038/s41380-022-01481-2 (PMC9718683; doi:10.1038/s41380-022-01481-2)
Supplement: Supplementary file 9 — Supplementary Tables [file 41380_2022_1481_MOESM9_ESM.docx]

**SUPPLEMENTARY TABLES**

**Supplementary Table 1** Antibodies, chemicals and materials used in this study.

| REAGENT or RESOURCE | SOURCE | IDENTIFIER |
| --- | --- | --- |
| Antibodies | | |
| Rabbit anti-AKT | Cell Signaling Technology | Cat#9272, RRID:AB_329827 |
| Rabbit anti-p-AKT Ser473 | Cell Signaling Technology | Cat#4060, RRID:AB_2315049 |
| Rabbit anti-p-AKT Thr308 | Cell Signaling Technology | Cat#13038, RRID:[AB_2629447](http://antibodyregistry.org/AB_2629447) |
| Rabbit anti-P85 | Cell Signaling Technology | Cat#4257, RRID:AB_659889 |
| Rabbit anti-p-PI3K P85 | Cell Signaling Technology | Cat#4228, RRID:AB_659940 |
| Rabbit anti-PTEN | Cell Signaling Technology | Cat#9188,  RRID:AB_2253290 |
| Rabbit anti-SGK1 | Abcam | Cat#ab59337, RRID:AB_945405 |
| Goat anti-rabbit, HRP-linked | Cell Signaling Technology | Cat#7074, RRID:AB_2099233 |
| Critical Commercial Assays | | |
| NucleoSpin miRNA | Macherey-Nagel GmbH & Co KG | Cat#740971 |
| Ovation SoLo RNA-Seq System, Mouse | NuGEN Technologies | Cat#0501-32 |
| Protein Quantification Assay | Macherey-Nagel GmbH & Co KG | Cat#740967 |
| ACD RNAscope Multiplex Fluorescent Reagent Kit v2 | ACDBio | Cat#323110 |
| TSA Plus Cyanine 5 | Perkin Elmer | Cat#NEL745001KT |
| Super Script IV First strand Synthesis System for qRT-PCR | Invitrogen | Cat#[18090050](https://www.thermofisher.com/order/catalog/product/18090050) |
| Criterion™ TGX Stain-Free™ Precast Gels | Bio-Rad | Cat#**5678045** |
| Nextera Index Kit | Illumina | Cat#FC-121-1011 |
| Nextera DNA Sample Preparation Kit | Illumina | Cat#FC-121-1030 |
| PowerUp SYBR Green Master Mix | Applied Biosystems by Thermo Fisher Scientific | Cat#A25742 |
| Deposited Data | | |
| Mouse reference genome NCBI BRCm38/mm10 | Genome Reference Consortium | N/A |
| RNA-seq dataset | This paper | GEO:GSE178210 |
| ATAC-seq dataset | This paper | GEO:GSE178210 |
| CUT&RUN dataset | This paper | GEO:GSE178210 |
|  |  |  |
| Experimental Models: Organisms/Strains | | |
| Mouse: CD1 strain | University clinics of Regensburg | N/A |
| Oligonucleotides | | |
| Primer | This paper | See table S1 |
| Antisense LNA GapmeRs | This paper, custom designed, Qiagen | Antisense LNA GapmeR Control, Negative A: #339516LG00000002-FFC;  NR_027651.2_5_5: #339524LG00213319-FFC;  NR_027651.2_5_3: #339511LG00213317-DDA;  NR_027651.2_5_1: #339511LG00213315-DDA;  NR_027651.2_5_2: #339511LG00213316-DDA |
| Software and Algorithms | | |
| Ballgown | Pertea et al., 2016 | https://github.com/alyssafrazee/ballgown |
| MACS2 | Zhang et al., 2008 | https://genomebiology.biomedcentral.com/articles/10.1186/gb-2008-9-9-r137 |
| HiSAT | Kim et al., 2015 | N/A |
| HOMER |  | <https://pubmed.ncbi.nlm.nih.gov/20513432/> |
| StringTie | Pertea et al., 2015 | N/A |
| DESeq2 | Love et al., 2014 |  |
| Prism 8 | GraphPad Software | https://www.graphpad.com/scientific-software/prism/ |
| TSE fear conditioning software | TSE Systems | N/A |
| JWatcher 1.0 |  | http://www.jwatcher.ucla.edu/ |
| Other | | |
| Meg3-ex10 probes – RNAscope probe “Mm-Meg3-O3” | ACDBio | Cat#573321 |

| **Name** | **Sequence 5'- 3'** | **Expected Length (bp)** |
| --- | --- | --- |
| m_Gapdh_F  m_Gapdh_R | AAGGGCTCATGACCACAGTC  CAGGGATGATGTTCTGGGCA | 111 |
| m_Hcrtr2_1F  m_Hcrtr2_1R | TTGTGGCTCTCATCGGGAAC  TTGGGTAAACTTCACCGCCC | 465 |
| m_Meg3_2F  m_Meg3_2R | GTCGCGAAGGGATGAGAGAG  GGCTGCTCTAGCCATTTCCA | 289 |
| m_Meg3_F (all variants)  m_Meg3_R | GTTGTGCTCAGGTTCCACGA  AACGTGTTGTGCGTGAAGTC | 184 |
| m_Meg3_short_1F  m_Meg3_short_1R | AGAAAAGAAGACTGAGGACCCC  CACAGGAAATGTGCAAGGCG | 250 |
| m_Nlrp5-ps_2F  m_Nlrp5-ps_2R | GGGACACCGCCCTGTTATATT  ACTGTTGTTTTCTGCAGGAGT | 93 |
| m_Plin4_2F  m_Plin4_2R | TCAGTGGAGGAGTGTGGTCA  TCATGTCTGTCATCTGGAAGGC | 304 |
| m_Sgk1_1F  m_Sgk1_1R | TCTTTTGGGCTCTTTCCGGG  TTGAGAGGGACTTGGCGGA | 273 |
| m_Sirt1_1F  m_Sirt1_1F | GGCCGCGGATAGGTCCATA  ACAATCTGCCACAGCGTCAT | 136 |

**Supplementary Table 2** Primer sequences

**Supplementary Table 3 Details of statistics for each experiment.** Abbreviations: SFC: social fear conditioning, SFC^+^: conditioned, SFC^-^: unconditioned

| **Figure 1** | | |
| --- | --- | --- |
| *SFC* | *Group effect (SFC)* | *Group x stimulus effect* |
| Social fear extinction (1C) | F(2, 59) = 174.6, p < 0.0001 | F(16, 472) = 44.44, p < 0.0001 |
| Relative Meg3-ex10 expression (1D) | Kruskall-Wallis H = 9.569, p = 0.0084 |  |
| Pearson Correlation (relative Meg3-ex10 expression vs. extinction success) (1E) | R = 0.46, p =0.0068 |  |
| **Figure 2** | | |
| *Relative Meg3-ex10 fold change* |  |  |
| 24 h after acquisition (SFC^+^/Ext^-^ vs. SFC^-^/Ext^-^) (2A, left panel) | T(16) = 2.428, p = 0.0274 |  |
| 3 h after after extinction (SFC^-^/Ext^+^ vs. SFC^+^/Ext^+^/suc vs. SFC^+^/Ext^+^/unsuc) (2A, middle panel)  Separate statistics SFC^+^/Ext^+^/suc vs. SFC^+^/Ext^+^/unsuc (2A, middle panel) | F(2, 22)= 2.134, p = 0.1422  T(13) = 2.163, p = 0.0498 |  |
| *Social Interaction (non-social vs social)/SFC* | *Group effect* | *Group x stimulus effect* |
| SFC with non-social stimuli (2B, middle panel) | F(2, 51) = 41.57, p < 0.0001 | F(16, 408)= 31.50, p < 0.0001 |
| Relative Meg3-ex10 expression (2B, right panel) | F(2, 45) = 8.145, p = 0.001 |  |
| Social interaction (2C, middle panel) | F(1, 14) = 28.27, p = 0.0001 | F(8, 112) = 7.908, p < 0.0001 |
| Relative Meg3-ex10 expression (2C, right panel) | T(14) = 1.508, p = 0.1537 |  |
| **Figure 3** | | |
| *SFC (control vs. Meg3-ex10 knockdown)* | *Group effect* | *Group x stimulus effect* |
| SFC extinction (3B) | F(3, 103) = 40.61, p < 0.0001 | F(24, 824) = 23.31, p < 0.0001 |
| Short-term recall Day 6(3C) | F(3, 36) = 1.438, p = 0.2478 | F(3, 36) = 0.5207, p = 0.6708 |
| Long-term recall Day 26 (3C) | F(3, 35) = 1.840, p = 0.1578 | F(3, 35) = 3.932, p = 0.0161 |
| Relative Meg3-ex10 expression (3D) | *Treatment effect*  F(1, 70) = 333.4, p < 0.0001 |  |
| SFC extinction (3G) | F(3, 20) = 12.97, p < 0.0001 | F(24, 160) = 8.185, p < 0.0001 |
| Relative Meg3-ex10 expression (3H) | *Treatment effect*  F(1, 2) = 0.3020, p < 0.2244 |  |
| Numbers of CS-US pairings (3I) | T(11) = 0.5754, p = 0.5766 |  |
| **Figure 4** | | |
| *Western blot analyses (SFC, 3 h after extinction)* | *Group effect (SFC)* |  |
| P85 phosphorylation (4B) | F(2, 20) = 4.251, p = 0.0289 |  |
| P85 protein expression (4C) | F(2, 20) = 0.7659, p = 0.4781 |  |
| AKT Thr308 phosphorylation (4D) | F(2, 20) = 0.8480, p = 0.4431 |  |
| AKT Ser 473 phosphorylation (4E) | F(2, 20) = 8.654, p = 0.0020 |  |
| AKT protein expression (4F) | F(2, 20) = 3.585, p = 0.0467 |  |
| PTEN protein expression (4G) | F(2, 20) = 0.0998, p = 0.9054 |  |
| *Western blot analyses (90 min after extinction, Meg3-ex10 knockdown experiments)* | *Group effect (SFC)* | *Group x Knockdown effect* |
| P85 phosphorylation (4I)  Control SFC^-^ vs. control SFC^+^  Control SFC^-^ vs. knockdown SFC^-^ | F(1, 31) = 1.500, p = 0.2298  T(14) = 2.531, p = 0.0240  T(15) = 1.998, p = 0.0642 | F(1, 31) = 3.575, p = 0.0680 |
| P85 protein expression (4J) | F(1, 31) = 0.02493, p = 0.8832 | F(1, 31) = 0.5780, p = 0.4529 |
| AKT Thr308 phosphorylation (4K) | F(1, 38) = 0.0132, p = 0.9091 | F(1, 38) = 0.9056, p = 0.3472 |
| AKT Ser 473 phosphorylation (4L) | F(1, 37) = 0.07185, p = 0.7902 | F(1, 37) = 0.1889, p = 0.6663 |
| AKT protein expression (4M) | F(1, 38) = 0.7726, p = 0.3850 | F(1, 38) = 0.06514, p = 0.7999 |
| PTEN protein expression (4N) | F(1, 36) = 0,7895, p = 0.3802 | F(1, 36) = 0,05246, p = 0.8201 |
| **Figure S1** |  |  |
| *RNA/protein expression* | *Group effect (SFC)* |  |
| Relative Nlrp5-ps expression (S1B) | Mann-Whitney U = 133; P < 0.5246 |  |
| Relative Hcrtr2 expression (S1C)  90 min  3 h | T(30) = 1.087, p < 0.2855;  Mann-Whitney U = 15; p < 0.0096 |  |
| Relative Plin4 expression (S1D) | Mann-Whitney U = 70; p < 0.3473 |  |
| Relative Sirt1 expression (S1E)  90 min  3 h | Mann-Whitney U = 111; p < 0.5391;  Mann-Whitney U = 27; p < 0.2268 |  |
| Relative Sgk1 expression 30 min (S1F) | F(2, 11) = 0.2945, p = 0.1885 |  |
| Relative Sgk1 expression 90 min (S1G) | F(2, 35) = 2.152, p = 0.1314 |  |
| Relative Sgk1 expression 3 h (S1H) | F(2, 21) = 1.809, p = 0.1885 |  |
| **Figure S2** |  |  |
| *Relative RNA expression* | *Group effect* |  |
| Relative Meg3 expression 90 min septum (S2C) | Kruskall-Wallis H = 0.9731, p = 0.6147 |  |
| Relative Meg3-short expression 90 min septum (S2D) | F(2, 38) = 0.1715, p = 0.8430 |  |
| Relative Meg3 expression 3 h septum (S2E) | F(2, 30) = 0.9378, p = 0.4027 |  |
| Relative Meg3-short expression 3 h septum (S2F) | F(2, 32) = 0.1351, p = 0.8741 |  |
| Relative Meg3-ex10 expression 90 min dorsal hippocampus (S2G) | F(2, 22) = 0.6311, p = 0.5414 |  |
| Relative Meg3-ex10 expression 90 min ventral hippocampus (S2H) | F(2, 22) = 1.577, p = 0.2291 |  |
| Relative Meg3-ex10 expression 3 h dorsal hippocampus (S2I) | F(2, 11) = 2.550, p = 0.1231 |  |
| Relative Meg3-ex10 expression 3 h ventral hippocampus (S2J) | F(2, 15) = 0.5460, p = 0.5903 |  |
| **Figure S4** |  |  |
| *Western blot analyses (SFC, 90 min after extinction)* | *Group effect (SFC)* |  |
| P85 phosphorylation (S4B) | F(2, 28) = 1.046, p = 0.3646 |  |
| P85 protein expression (S4C) | F(2, 28) = 2.010, p = 0.1529 |  |
| AKT Thr308 phosphorylation (S4D) | F(2, 30) = 0.1168, p = 0.8901 |  |
| AKT Ser 473 phosphorylation (S4E) | F(2, 30) = 0.07354, p = 0.9293 |  |
| AKT protein expression (S4F) | F(2, 29) = 1.725, p = 0.1960 |  |
| PTEN protein expression (S4G) | F(2, 33) = 0.7878, p = 0.4632 |  |
| **Figure S5** |  |  |
| *SFC (control vs. Meg3-ex10 knockdown)* | *Group effect (SFC)* | *Group x stimulus effect* |
| SFC extinction (S5A) | F(2, 6) = 67.88, p < 0.0001 | F(16, 48) = 24.05, p < 0001 |
| SFC extinction (S5B) | F(1, 4) = 2.219, p = 0.2105 | F(8, 32) = 1.735, p = 0.1282 |
| SFC extinction (S5C) | F(2, 6) = 70.58, p < 0.0001 | F(16, 48) = 27.05, p < 0001 |
| SFC extinction (S5D) | F(1, 4) = 2.102, p = 0.2207 | F(8, 32) = 1.449, p = 0.2150 |
| **Figure S2** |  |  |
| *Relative RNA expression* | *Group effect* | *Group x Knockdown effect* |
| Relative Auts2 expression 90 min (S6A) | F(2, 40) = 7.31, p = 0.002 |  |
| Relative Auts2 expression 3h (S6B) | F(2, 37) = 0.723, p = 0.49 |  |
| Relative Auts2 expression 5h (S6C) | Kruskall-Wallis H = 8.836, p = 0.012 |  |
| Relative Auts2 expression 90min, Meg-ex10 knockdown experiments (S6D) | F(1, 29) = 0.5037, p = 0.4835 | F(1, 29) = 0.0594, p = 0.8090 |
| Relative Dclk3 expression 90 min (S6E) | Kruskall-Wallis H = 2.46, p = 0.2922 |  |
| Relative Dclk3 expression 3h (S6F) | Kruskall-Wallis H = 6.679, p = 0.0355  Dunn’s multiple comparison test (SFC^-^/Ext^+^ vs. SFC^+^/Ext^+^/suc): p = 0,0602 |  |
| Relative Dclk3 expression 5h (S6G) | Kruskall-Wallis H = 3.165, p = 0.2055 |  |
| Relative Dclk3 expression 90min, Meg-ex10 knockdown experiments (S6H) | F(1, 29) = 0.5277, p = 0.4734 | F(1, 29) = 1, p = 0.3256 |
